# Stable integrant-specific differences in bimodal HIV-1 expression patterns revealed by high-throughput analysis

**DOI:** 10.1371/journal.ppat.1007903

**Published:** 2019-10-04

**Authors:** David F. Read, Edmond Atindaana, Kalyani Pyaram, Feng Yang, Sarah Emery, Anna Cheong, Katherine R. Nakama, Cleo Burnett, Erin T. Larragoite, Emilie Battivelli, Eric Verdin, Vicente Planelles, Cheong-Hee Chang, Alice Telesnitsky, Jeffrey M. Kidd

**Affiliations:** 1 Department of Human Genetics, University of Michigan Medical School, Ann Arbor, Michigan, United States of America; 2 Department of Microbiology and Immunology, University of Michigan Medical School, Ann Arbor, Michigan, United States of America; 3 West African Centre for Cell Biology of Infectious Pathogens (WACCBIP) and Department of Biochemistry, Cell & Molecular Biology, University of Ghana, Legon, Greater Accra Region, Ghana; 4 Department of Pathology, University of Utah, Salt Lake City, Utah, United States of America; 5 Department of Medicine, University of California San Francisco, San Francisco, California, United States of America; 6 Buck Institute for Research on Aging, Novato, California, United States of America; Miller School of Medicine, UNITED STATES

## Abstract

HIV-1 gene expression is regulated by host and viral factors that interact with viral motifs and is influenced by proviral integration sites. Here, expression variation among integrants was followed for hundreds of individual proviral clones within polyclonal populations throughout successive rounds of virus and cultured cell replication, with limited findings using CD4+ cells from donor blood consistent with observations in immortalized cells. Tracking clonal behavior by proviral “zip codes” indicated that mutational inactivation during reverse transcription was rare, while clonal expansion and proviral expression states varied widely. By sorting for provirus expression using a GFP reporter in the *nef* open reading frame, distinct clone-specific variation in on/off proportions were observed that spanned three orders of magnitude. Tracking GFP phenotypes over time revealed that as cells divided, their progeny alternated between HIV transcriptional activity and non-activity. Despite these phenotypic oscillations, the overall GFP+ population within each clone was remarkably stable, with clones maintaining clone-specific equilibrium mixtures of GFP+ and GFP- cells. Integration sites were analyzed for correlations between genomic features and the epigenetic phenomena described here. Integrants inserted in the sense orientation of genes were more frequently found to be GFP negative than those in the antisense orientation, and clones with high GFP+ proportions were more distal to repressive H3K9me3 peaks than low GFP+ clones. Clones with low frequencies of GFP positivity appeared to expand more rapidly than clones for which most cells were GFP+, even though the tested proviruses were Vpr-. Thus, much of the increase in the GFP- population in these polyclonal pools over time reflected differential clonal expansion. Together, these results underscore the temporal and quantitative variability in HIV-1 gene expression among proviral clones that are conferred in the absence of metabolic or cell-type dependent variability, and shed light on cell-intrinsic layers of regulation that affect HIV-1 population dynamics.

## Author summary

Very few HIV-1 infected cells persist in patients for more than a couple days, but those that do pose life-long health risks. Strategies designed to eliminate these cells have been based on assumptions about what viral properties allow infected cell survival. However, such approaches for HIV-1 eradication have not yet shown therapeutic promise, possibly because many assumptions about virus persistence are based on studies involving a limited number of infected cell types, the averaged behavior of cells in diverse populations, or snapshot views. Here, we developed a high-throughput approach to study hundreds of distinct HIV-1 infected cells and their progeny over time in an unbiased way. This revealed that each virus established its own pattern of gene expression that, upon infected cell division, was stably transmitted to all progeny cells. Expression patterns consisted of alternating waves of activity and inactivity, with the extent of activity differing among infected cell families over a 1000-fold range. The dynamics and variability among infected cells and within complex populations that the work here revealed has not previously been evident, and may help establish more accurate correlates of persistent HIV-1 infection.

## Introduction

Early in the HIV-1 replication cycle, a DNA intermediate integrates into the host cell’s genome. HIV-1 replication ordinarily progresses into its late phases, with viral gene expression, virion production, and cell death. However, some proviruses can remain dormant upon integration. In patients, the resulting latently infected cells persist throughout antiretroviral treatment, and their sporadic reactivation can lead to virus rebound after antiretroviral cessation.

This source of persistent virus is called the latent reservoir and is believed to consist largely of transcriptionally silent proviruses integrated into resting memory T cells [1] [2] [3]. Experimentally, infectious virus can be produced by T lymphocytes from such patients when the cells are activated or treated with certain chromatin remodeling drugs *ex vivo*. These observations inspired “shock and kill” HIV cure strategies, which involve pharmacologically inducing provirus expression to promote the recognition and clearance of latently infected cells [4] [5]. However, while intervention that reactivates silenced proviruses can activate HIV-1 gene expression in cell culture models of latency, such treatments have thus far failed to fulfill their promise in the clinic, suggesting much remains to be learned about the establishment and maintenance of the latent reservoir [6] [7] [8].

HIV-1 gene expression requires sequence motifs within proviral sequences that specify nucleosome positioning and allow HIV-1 to respond to host factor differences among infected cell types [9] [10] [11]. HIV-1 has a marked preference for integration in transcriptionally active genome regions [12, 13], and certain host chromatin binding factors as well as nuclear architecture further bias the distribution of integration sites [14, 15]. Integration sites influence HIV gene expression [16] [17] [18] [19], and it has been postulated that integration sites may affect the odds of a provirus establishing long-lived latency [20]. Differences in HIV-1 expression due to integration site features likely influence the extent to which cells survive and proliferate after HIV-1 integration, and in turn contribute to the expression profile of persistent HIV-1 [21].

Recent work with patient samples has demonstrated that for at least some suppressed patients, residual provirus-containing cells are polyclonal yet dominated by a limited number of clonal subsets [22], and similar observations of clonal expansion have been made during HIV-1 infection of humanized mice [23]. Thus, the integration sites represented in persistent proviruses are probably a limited subset of the spectrum initially generated [21].

Recent evidence indicates that latent proviruses differ in the extents to which they can be reactivated, and that a large majority of cells harboring latent proviruses may be refractory to our current arsenal of reactivation agents [24, 25]. Work using dual color reporter viruses in primary cells has shown that proviruses differ in their reactivation potential depending on their sites of integration, with chromatin context as maintained within the confines of the nucleus being a significant contributing factor [25]. Additional work monitoring HIV-1 expression in individual cells has questioned the earlier view that complete proviral silencing is necessary for infected cell persistence during antiretroviral therapy [26, 27].

The majority of proviruses detectable in suppressed patients are replication defective [26, 28]. Although such proviruses are incapable of rekindling infection, emerging evidence suggests they can be expressed and may contribute to pathogenesis [26, 29].

In this study, we developed a high throughput approach to monitor cellular and viral progeny of individual integration events within complex populations, and used it to address the frequency of defective provirus formation and the extent to which provirus integration sites affect provirus expression levels. Initial work was performed using transformed cell lines, where selective pressures and variation of intracellular factors should be lower than in primary cells, with additional experiments performed in CD4+ lymphocytes from donor blood. Examining the extent of expression variation within and among cellular progeny of large panels of individual HIV-1 integration events indicated that in all tested cell types, epigenetic differences among proviral clones led to the establishment of distinct heritable patterns of HIV-1 gene expression.

## Results

### Nearly 90% of zip coded proviruses supported a second round of replication

**Fig 1:**
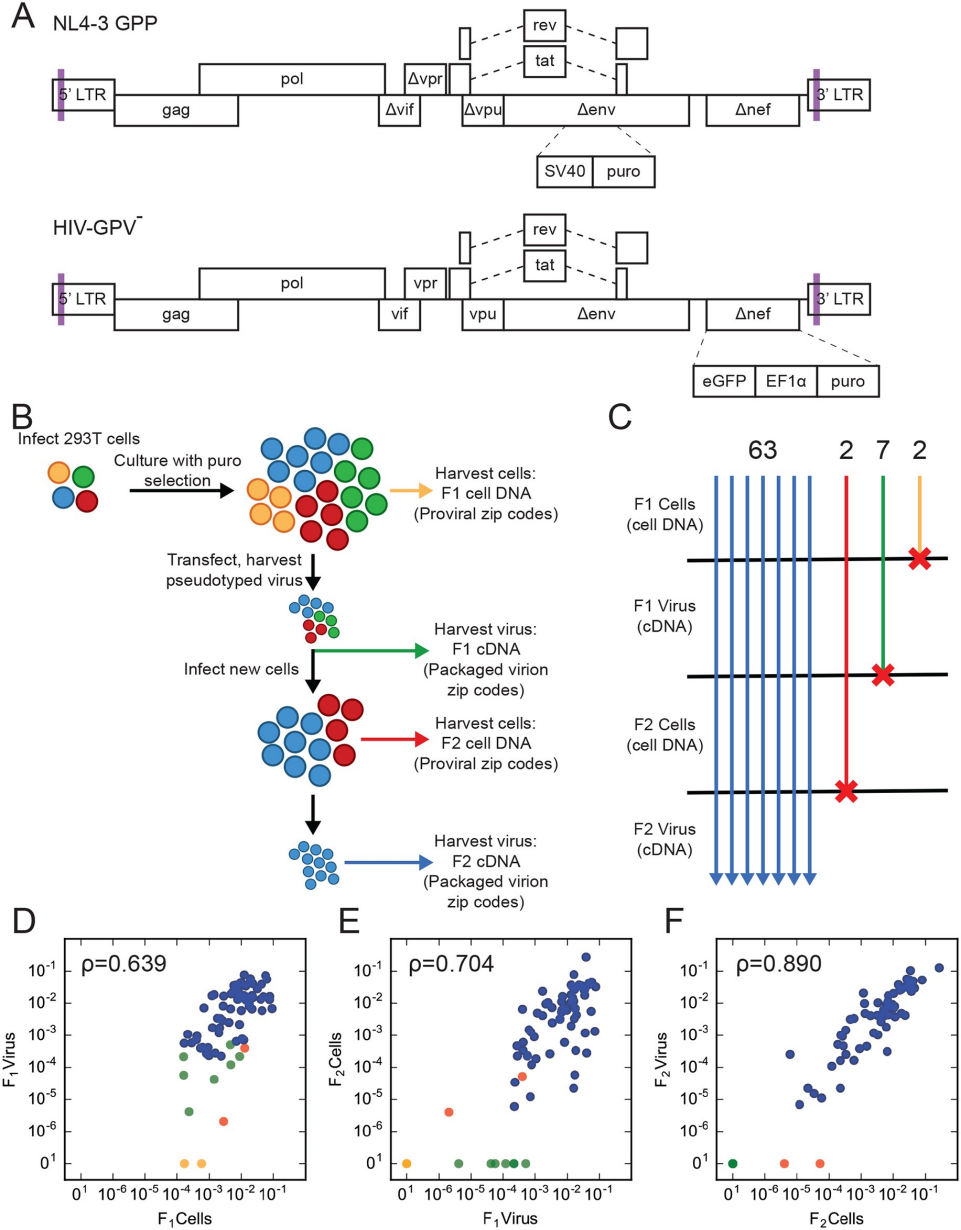
Monitoring proviral replication competence across generations. (A) Schematic illustrations of the vectors used in this paper. Lavender bars represent the sites of randomized sequence insertions. Features and construction are described in Materials and Methods. (B) Schematic of the experimental flow of the replication competence experiment, depicting the analysis of genomic DNA and viral cDNA harvested from the F_1_ and F_2_ generations. Each color represents members of a clone: on initial integration, one cell (represented by a circle) per color. The loss of colors over the course of the experiment represents predicted outcomes of mutational inactivation. (C) Summary of the number of independent zip codes detectable at different stages of the experiment. A total of 63 zip codes were detected in all four pools. The number of clones present at the indicated stage but not later are indicated at the top. (D, E, F) Scatter plots of zip code read proportions across indicated stages of the experiment, as outlined in (B). Each clone is represented by a single point, colored to reflect that clone’s persistence based upon the progression pattern depicted in (C). The Spearman correlation for each comparison is given.

We developed a system to identify individual HIV-1 proviral lineages within polyclonal populations, track proviral gene expression, and monitor replication properties of individual cell clones and their viral progeny. To achieve this, NL4-3 strain-derived vectors that encoded Gag, Pol, Tat, Rev and a puromycin reporter (pNL4-3 GPP [30]; Fig 1A) were modified to each contain a unique 20-base randomized sequence tag. Once integrated, these were called “zip coded” proviruses because the tags reported provirus locations. Tags were inserted into the upstream edge of U3, downstream of integrase recognition sequences and upstream of the site of nuc0 nucleosome binding [11]. Vector RNAs were transcribed from uncloned DNA template libraries generated by *in vitro* assembly without amplification by plasmid replication. High throughput sequencing confirmed that the tag complexity of the starting library vastly exceeded the analyzed provirus population size (S1 Fig). Because the process of reverse transcription duplicates U3, each progeny provirus contained the same randomized tag in both LTRs, and each provirus’s tags differed from those in every other integrant.

To validate this approach, adherent 293T cells were transduced at a very low multiplicity of infection (<0.00005) and the randomized regions amplified from ten individual puromycin-resistant colonies were sequenced. The results showed that no two colonies contained the same 20-mer (S1 Table).

An initial pooled-clone pilot study was then performed, which addressed the frequency of defective provirus formation during a single replication round (Fig 1B). 71 well-separated puromycin-resistant colonies were combined to generate an F_1_ cell pool. After expansion, pseudotyped virions (“F_1_ virus”) generated from F_1_ cells were used to infect fresh 293T cells. Because the number of colonies pooled to generate the F_2_ cell pool—roughly 1000—was significantly greater than the F_1_ pool’s zip code complexity, any infectious zip code present in the F_1_ pool was predicted to generate multiple F_2_ integrants.

The ability of each F_1_ provirus to complete a second replication round was addressed by comparing F_1_ and F_2_ virion cDNA and F_1_ and F_2_ cell DNA zip codes using high throughput sequencing (Fig 1B). How zip codes were analyzed and quantified is described in Materials and Methods, and included ranking zip codes based on sequencing read frequencies, beginning with the most abundant. F_1_ pool cells were found to contain 74 unique zip codes, which accounted for 99.87% of total sequencing reads (S2 Fig). Although the possibility of dual infection cannot be ruled out, the low multiplicity of infection used here suggested the discrepancy between this value and the 71 colonies visualized was likely due to miscounting double colonies as single expanded clones. Because 65 out of the 74 zip codes found in F_1_ cell DNA were also observed in the F_2_ cell library, these 65 (88% of F_1_ cell zip codes) unambiguously represented proviruses capable of completing a second round of replication (Fig 1C). The remaining 9 zip codes were candidate non-infectious proviruses. Two of these were detectable in the F_1_ cell library but not in F_1_ virus cDNA, thus displaying the phenotype predicted for genomes mutationally inactivated during reverse transcription. If a first-round provirus could assemble but not replicate, its zip code might be detectable in F_1_ virus but not F_2_ cells. The seven remaining zip codes were candidates for this class of defective proviruses (green lines in Fig 1C).

The properties of two of the 65 infectious clones were initially enigmatic. The number of colonies pooled to make the F2 library suggested it contained roughly twenty re-transduced copies of each F1 zip code. Based on how frequently replication competence was maintained after the first round of replication, any fully infectious F_1_ provirus was expected to display a roughly 90% second-round success rate. Thus, the likelihood that all ~20 sibling F_2_ progeny of any infectious F_1_ provirus would be defective seemed exceptionally low. However, among the 65 replication-competent zip codes in the F_2_ cell library, two were not observed in F_2_ virus RNA.

### Integrant clone expansion and provirus expression levels varied widely among zip coded 293T cell clones

To address whether the absence of two F_2_ cell zip codes from the F_2_ virus library might reflect a population bottleneck, the number of sequencing reads associated with each zip code was compared within and across libraries. Unexpectedly, reads per zip code were observed to vary over three orders of magnitude within the F_1_ cell library (Fig 1D). Although variation in the expansion rates of provirus-containing cells have been reported previously [31], the wide range in cell clone sizes observed here had not been anticipated.

Clone-specific differences in the amount of virus released per cell were also observed (Fig 1D, y axis). When normalized to the number of F_1_ cells harboring a given zip code, differences in virion release per cell spanned two orders of magnitude or more. Because of this, zip code abundance in the F_1_ cell and F_1_ virus libraries were only moderately correlated (Fig 1D) (Spearman ρ = .639, p = 8.7*10^−10^). In contrast, the correlation between cell count and virion production was strong in the F_2_ generation (Spearman ρ = .890, p = 3.75*10^−23^) where each zip code was polyclonal (Fig 1F), suggesting that virus-per-cell ratios were fairly consistent when averaged across many cell clones.

Looking specifically at sequencing read data for the two F_2_ cell zip codes that were missing from F_2_ virus revealed that these lineages were scarce in both the F_1_ virus and in F_2_ cells (red points, Fig 1E). Similarly, read frequency trends for the seven F_1_ zip codes not observed in F_2_ cells (green points, Fig 1D) suggested that population bottlenecks, and not loss of infectivity, may account for the absence of some of these in F_2_ cells.

The pilot studies above validated assessing multiple proviral lineages within cultured cell populations by tracking read counts in high throughput sequencing libraries. However, because the cells were not physically cloned, it remains possible that experimental procedures may have introduced unintended variation and skewed read counts. Nonetheless, amplicons were the same length for all library members and were sequenced at apparently similar frequencies within the starting virus pool (S1 Fig). This suggests minimal bias in the amplification and sequencing of zip codes, which—paired with the practice of performing all high throughput library preparation and cell sorting experiments in at least duplicate in the experiments below—provides general support for the assumption applied below: namely, that zip code read frequencies in sequencing libraries reflected the abundance of that zip code within the cell population used to generate the library.

### Clonal expansion in Jurkat cells

Larger zip coded integrant populations were then established using Jurkat cells. The vector in these experiments (HIV GPV^-^) expressed all HIV-1 genes except *env*, *nef*, and *vpr*, contained GFP in the *nef* open reading frame, and expressed a selectable marker (for puromycin resistance) from a secondary, internal promoter, as has been done in previous strategies [32] (Fig 1A, lower construct). Selective concentrations of puromycin were applied briefly, and cells were subsequently maintained without drug. Cell pools infected with differing amounts of virus were analyzed by high throughput sequencing, and one of these pools, which was determined to contain roughly 1,000 zip coded clones, was used in subsequent studies.

Sequencing duplicate aliquots of this pool revealed that many zip codes were shared in both replicates, but lower abundance zip codes were sampled unevenly. To better address the complexity of the pool and differential clone expansion, ten technical replicates were combined to provide evidence for 706 zip codes, which together accounted for 97.8% of the total reads. Based on zip code sequencing read frequencies (S3 Fig), the pool displayed clonal abundances spanning over two orders of magnitude, with the most prevalent half-dozen zip codes each accounting for >1% of the total reads while the lower 10% of the 706 zip codes each contained <0.01% of the total reads.

### Significant clone-by-clone differences in HIV-1 expression in both Jurkat and primary cells

**Fig 2:**
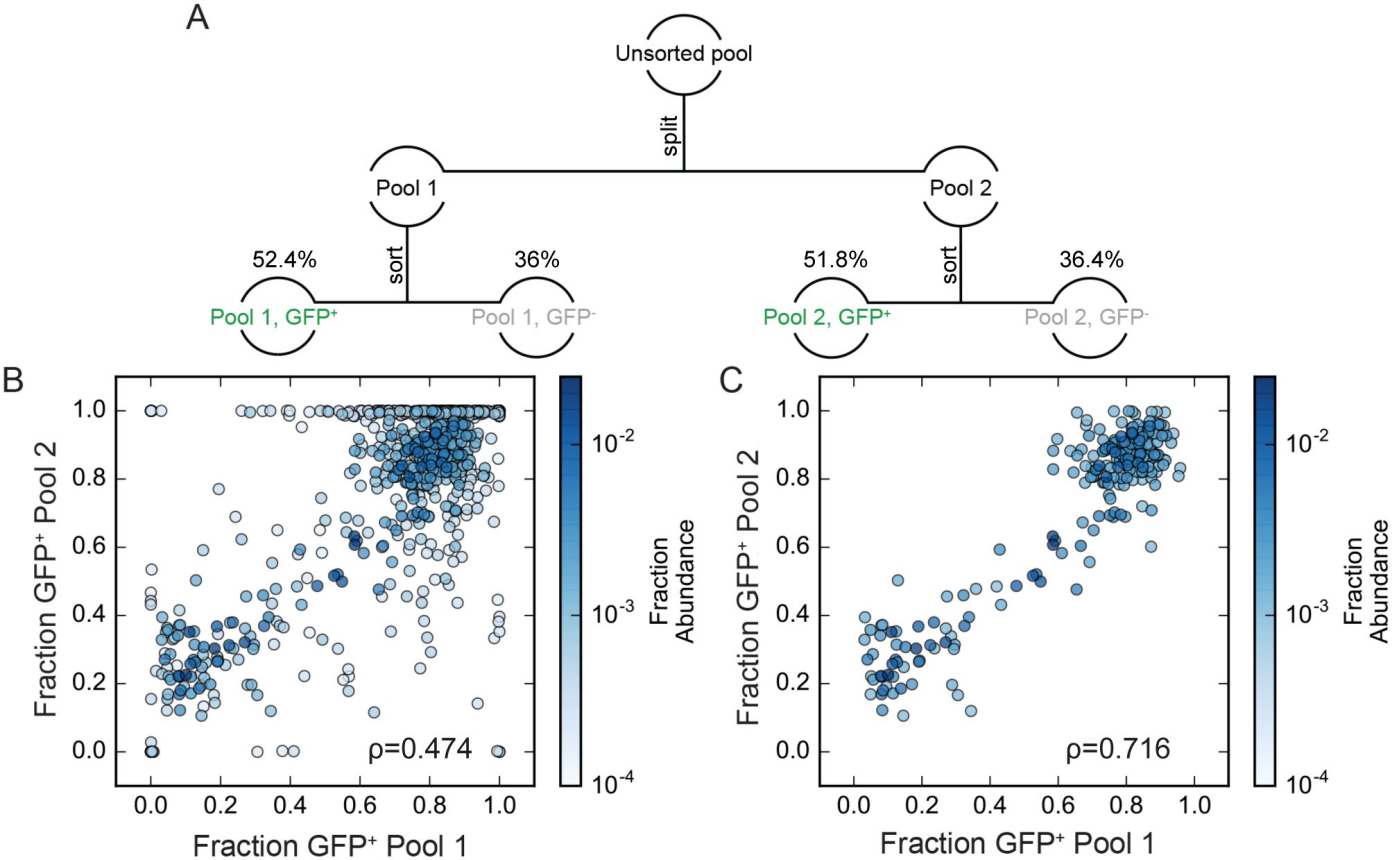
GFP+ proportions for independent clonal lines within a complex population. (A) Schematic description of the cell pool splitting and sorting procedures performed. GFP+ proportions were determined as described in the text. (B) comparison of fraction GFP+ determined for each zip code in Pool 1 and Pool 2. Each point represents a single zip coded cell clone. Individual clones are colored based on their fractional abundance in the original unsorted pool as indicated by the color bar on the right side of the panel. (C) as in (B), but with data for the less abundant clones removed to show only the 225 zip codes with fractional abundance > 0.001.

Detecting GFP by flow cytometry allowed binary (on/off) monitoring of LTR expression in individual cells, and work here used GFP as a surrogate for HIV-1 gene expression. Portions of the total Jurkat pool characterized above, designated Pools 1 and 2, were independently sorted into GFP positive “GFP+” and negative “GFP-” sub-pools (Fig 2A; S4 Fig shows how sorting was gated). As a control, cells were also analyzed by FACS based on their p24 content using an anti-p24 antibody, and a strong correlation between GFP expression and p24 content was observed (S5 Fig).

An expression value termed the “GFP+ proportion” was determined for each zip coded clone. GFP+ proportions were calculated by dividing the read frequency of each zip code in GFP+ sorted cells by the sum of the abundance of that zip code in GFP+ and GFP- sorted cells after weighting values to reflect the proportions of total cells in the GFP+ and GFP- sub-pools. A sample calculation is provided in Materials and Methods. Consistent with clonal variation in virus release per cell observed in the pilot experiment above, GFP+ proportions differed substantially among Jurkat cell clones, with the GFP+ proportions of individual clones ranging from >99% to <1% (Fig 2).

To test if the broad range of clones’ GFP+ proportions reflected clone-specific properties or were a result of sampling, we compared data for duplicate experimental samples, with the GFP+ proportions calculated for each zip code in Pool 1 compared to those independently determined for Pool 2. As shown in Fig 2B, when GFP+ proportion data were plotted against each other, most clones displayed similar values, suggesting that each clone possessed a distinct GFP+ proportion that was not defined by sampling (Spearman ρ = 0.474, p = 8.23*10^−40^ for the 688 zip codes detected in each pool). GFP+ proportions were particularly well correlated for the most abundant zip codes (Fig 2C, Spearman ρ = 0.716, p = 1.22*10^−36^ for the 225 zip codes with fractional abundance > 0.001 in the parental pool), suggesting that at least 200 clones were sufficiently abundant in the total population to be reproducibly well sampled in repeated sub-pools.

The experiments above were performed with cell lines, where within-experiment differences in environment and *trans*-acting factors should be minimized [33]. In an initial test of whether primary cells also displayed integrant-specific differences in our system, CD4+ cells were isolated from donor blood, stimulated, and transduced with VSV-G pseudotyped zip coded GPV^-^ (S6 Fig). Six days post infection, the cells were divided into 2 sub-pools that were each sorted into GFP- and GFP+ cell fractions, and the GFP+ proportions of individual clones in each sub-pool were compared. Three independent experimental repetitions were performed, and the approaches are described in Materials and Methods and S6 Fig. The results showed that in these primary cell experiments, most zip codes were represented by very few sequencing reads, possibly due to variation in primary cell division rates and to the retention of significant amounts of unintegrated viral DNA during the relatively short duration of primary cell propagation. Additionally, the number of clones that were sampled sufficiently to meet inclusion criteria (that the clone was detectable with fractional abundance > 0.0001 in each sub-pool) was low. Nonetheless, significant correlations were observed when the GFP+ proportions for these primary cell zip codes were calculated for each independently analyzed sub-pool and values for the two replicate sorts within each experimental repetition were plotted against one another (S6 Fig), indicating that the provirus-containing progeny of primary cells have clonal differences in HIV-1 gene expression levels.

### GFP+ proportions of clones are a stable, heritable phenotype

**Fig 3:**
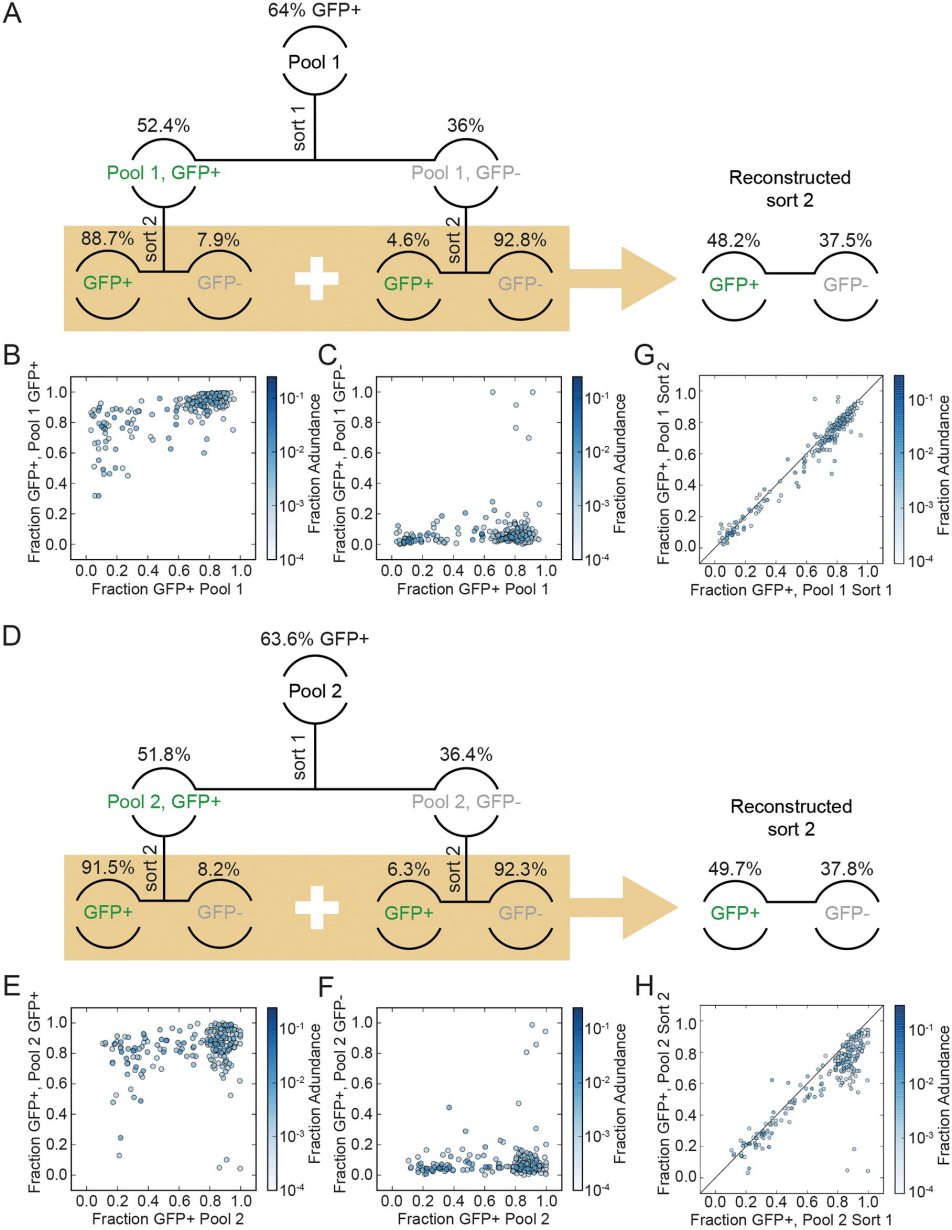
GFP+ proportions of passaged and re-sorted GFP+ and GFP- cell pools. (A) Depiction of the cells’ passaging and sorting scheme, with the initial sorted pools characterized in Fig 2 at the top, followed by the re-sorted sub-pools analyzed here. The % GFP+ above Pool 1 or Pool 2 in (A) or (B) respectively represents the % GFP+ cells in that Pool prior to sorting. The percentages listed for the sorted cells below that indicate the proportion of the unsorted pool that were sorted into the indicated samples, after gating as described in S4 Fig. (B) Analysis of zip codes that sorted GFP+ in Pool 1. Y axis indicates the GFP+ proportions determined in the second sort (within beige shaded box; this was a re-sorting of the sub-pool that had sorted GFP+ in the first sort and had been passaged separately for > I week) and X axis is GFP+ proportions from Pool I first sort (eg: Fig 2C X axis) (C) Analysis of zip codes that sorted GFP- in Pool 1. Y axis indicates the GFP+ proportions determined in the second sort (within beige shaded box; this was a re-sorting of the sub-pool that had sorted GFP- in the first sort and had been passaged separately for > I week) and X axis is GFP+ proportions from Pool I first sort (eg: Fig 2C X axis) (D, E, F) Analysis performed as in Fig 3A, 3B and 3C, for zip codes in Pool 2. (G) Stability of GFP+ proportions in Pool 1 over time. GFP+ proportions determined in the first sort (Fig 3 data) plotted against the reconstructed second sort, as assessed by comparing GFP+ proportions for each zip code derived from data in Pool 1 at the second sort (Y axis, data from panels B and C) vs the first sort (Fig 2C X axis). Second sort GFP+ proportions were reconstructed by weighting the GFP+ and GFP- sub-pool values determined in panels A and B as described in Materials and Methods. (H) Stability of GFP+ proportions in Pool 2 over time, performed as described in panel G for Pool 1.

Longitudinal studies were then performed with the zip coded Jurkat pool established in Fig 2A, to monitor GFP+ proportions throughout cell generations. After sampling for sequencing library preparation, aliquots of the GFP+ and GFP- sub-pools of both Pool 1 and Pool 2 were passaged separately for an additional 8 to 9 days, at which time point each of these four sub-pools were again sorted by FACS (Fig 3A and 3D). The results showed that the cellular descendants of Pool 1 and Pool 2 GFP+ sub-pools did not all remain GFP+ (Fig 3B and 3E), nor did the descendants of the GFP- sub-pools remain all GFP- (Fig 3C and 3F). Instead, some cells from each sub-pool had switched expression phenotypes during passaging. This suggested that the HIV-1 expression pattern in any individual cell was not stably inherited by all of its progeny, but that instead expression “flickered” (alternated between LTR expression and silencing) during cell propagation.

Integrant specific, intrinsic rates of expression that are maintained across cell generations have previously been reported for basal expression from the HIV-1 promoter [13]. To test whether or not the expression patterns studied here also were stable over time, the GFP+ proportions determined for the GFP+ or GFP- pools in the second sort were combined after weighting to reconstitute the original unfractionated population (reconstructed second sort pools in Fig 3A and 3D; described in Materials and methods). The GFP+ proportions in Pool 1 and Pool 2 at the time of the first sort were then compared to the GFP+ proportions of the reconstructed pools at the time of the second sort (Fig 3G and 3H). Consistent with the stable inheritance of clone-specific intrinsic expression patterns, here the data indicated that the weighted GFP+ proportions for each integrant following the second sort showed a strong correlation with its GFP+ proportion in the first sort (Spearman ρ = 0.939, p = 1.1*10^−105^ for Pool 1 and 0.806, p = 1.2*10^−52^ for Pool 2; Fig 3 first sort *vs*. reconstructed second sort GFP+ proportion values).

### Correlates of integration site features and provirus activity

**Fig 4:**
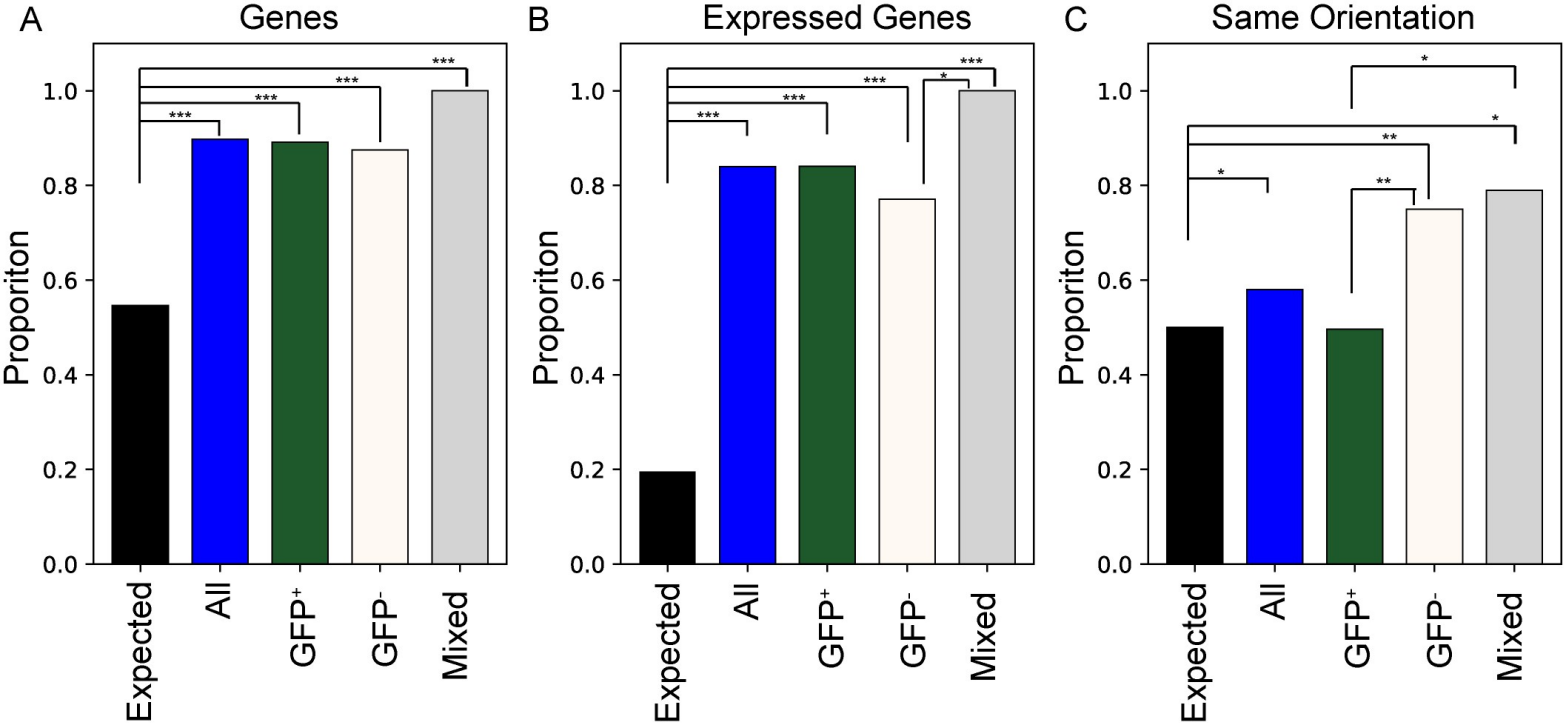
Integration site features. Integration site properties are shown for each zip code. In each panel, the “expected” bar shows the proportions that would arise if integration occurred uniformly at random positions throughout the genome, “all” represents proportions for all examined zip coded integrants, and GFP+, GFP-, and Mixed represent zip codes assigned to the mostly GFP+, mostly GFP-, or mixed clones, as described in the text. (A) Comparison of proportions of each category of integrants that resides in annotated genes. (B) Proportions within genes annotated as expressed in Jurkat cells [36] (C) Comparison of proportions of each category of integrants that resides in the same orientation as gene transcription. Statistically significant pair-wise differences are indicated by bracket lines and an asterisk symbol with * indicating p<0.05, ** p< 0.01, and *** p< 0.001. Results for (A) and (B) were determined by permutation while results for (C) are based on a binomial test. Nominal p-values are indicated without correction for the number of tests performed.

Integration site features were compared to address whether these features affected the viral gene expression patterns observed here. Integration sites were determined using a linker-mediated nested PCR strategy applied to genomic DNA from the original unsorted Jurkat pool. Primers were designed so that sequencing reads included integration site sequences and U3 resident zip codes. Initial analysis indicated variable rates of assignment of a single zip code to multiple genomic locations, likely reflecting the formation of chimeric molecules during PCR [34, 35]. We therefore implemented an algorithm that removed minor assignments presumed to be PCR artifacts and selected abundant, redundantly implicated integration sites. This strategy assigned genomic location to each of the 225 high abundance zip codes (S2 Table). As expected [36], integrants were substantially enriched for annotated genes and genes expressed in Jurkat cells (Fig 4A and 4B), with 58% having the same orientation as the intersecting transcript (109 of 188 that intersect with single genes, p = 0.034, binomial test).

To search for factors that may affect set point expression levels, we assigned each of the 225 zip codes to one of three classes based on their balance of bimodal expression: those with a GFP+ proportion of at least 0.6 in both pools (‘mostly GFP+’; 157 clones), those with a GFP+ proportion less than 0.4 in both pools (‘mostly GFP-’; 48) and those with mixed levels of GFP expression (‘mixed’; 20). Ignoring integrants that intersect with no genes or with genes having overlapping expression in divergent directions, we found no orientation preference for the ‘mostly GFP+’ integrants (65 of 129 with single intersection have same orientation; p = 0.99 binomial test) (Fig 4C), whereas both the mostly GFP- and mixed populations were enriched for integration in the same orientation as gene transcription (30 out of 40; p = 0.002 and 15 out of 19; p = 0.019). The GFP+ proportion of each integrant had a strong negative correlation with original abundance in the pool (Spearman ρ = -0.289, p = 1.08*10^−5^).

**Fig 5:**
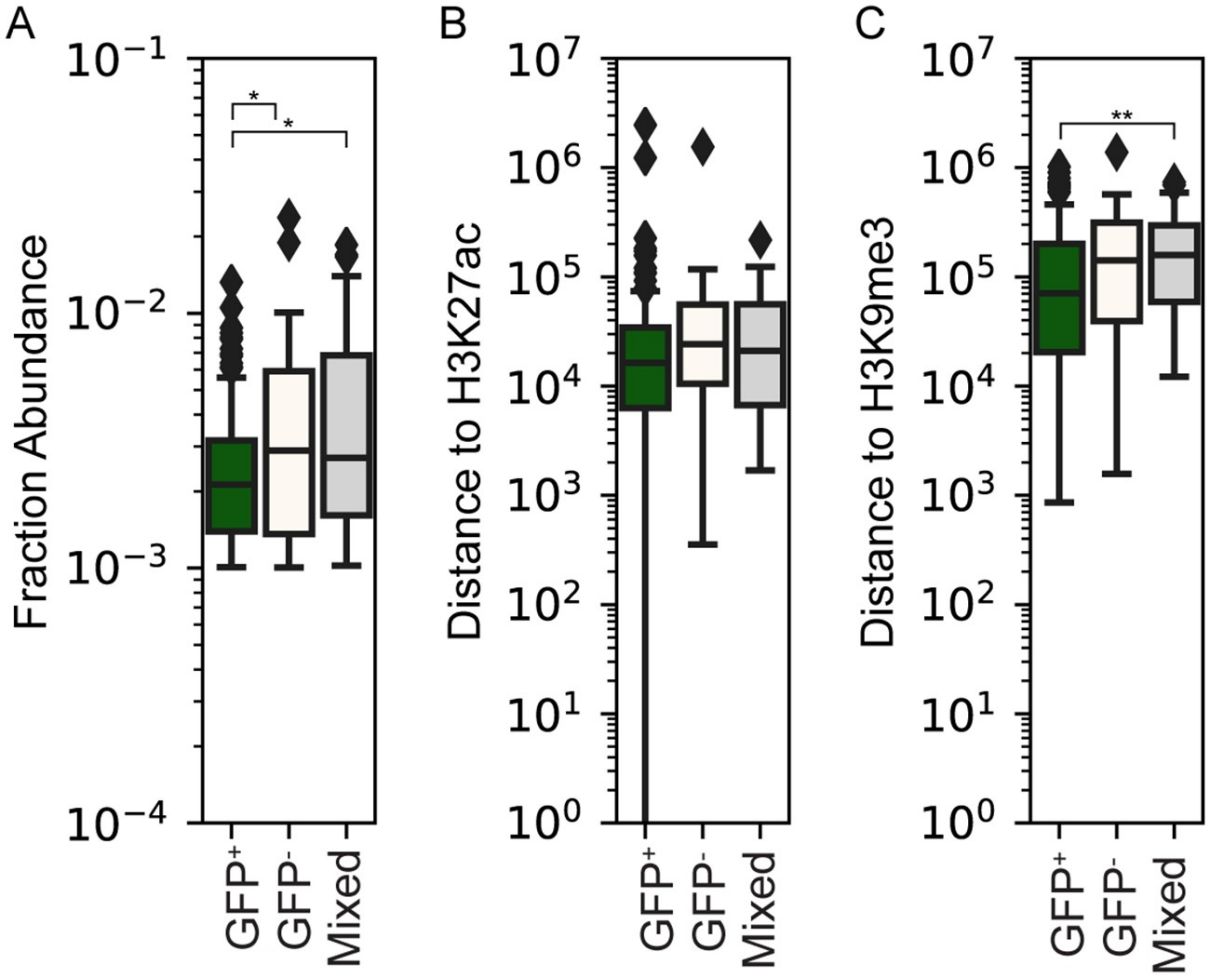
Correlations between GFP+ proportions and mapped epigenetic features. Each of the 225 zip codes were binned into one of three categories (mostly GFP+, mostly GFP-, or mixed, as described in the text). (A) box plots show the fractional abundance of each zip code residing in that category of clones, as determined in the unsorted Jurkat pool (Fig 2 data). (B) distances to H3k27ac and (C) H3k9me3 peaks, respectively, for the mostly GFP+, mostly GFP-, and mixed expression pattern zip codes. For each box plot the median and interquartile range is depicted. Pairwise comparisons with significant differences based on a Mann-Whitney U two sided test are indicated, * = p<0.5, ** = p<0.01. Nominal p-values are indicated without correction for the number of tests performed.

We additionally compared the distance of integrants to enhancer associated (H3K27ac) and repressive (H3K9me3) chromatin marks previously determined in Jurkat cell lines [37, 38] (Fig 5), again based on their balance of bimodal expression. Distance to H3K27ac peaks had a negative but non-significant correlation to GFP+ proportion (Spearman ρ = -0.105, p = 0.118). Distance to existing H3K9me3 repressive marks in Jurkat cells was also negatively correlated with GFP+ proportion (Spearman ρ = -0.195, p = 0.0034). Thus, these results conflictingly showed that integrants with higher GFP expression states were on average closer to both existing repressive and enhancer chromatin marks. Comparing the range of values across classes revealed the modest nature of these enrichments (Fig 5), with mostly GFP+ and mostly GFP- clones having a significant difference in original clone abundance (p = 0.044, Mann Whitney U 2-sided test) and a nearly significant difference in distance to H3K9me3 peaks (p = 0.07, Mann Whitney U 2-sided test), while the distance to H3K9me3 peaks was significantly different between the GFP+ and ‘mixed’ classes (p = 0.004, Mann Whitney U 2-sided test).

## Discussion

Here, persistence and HIV-1 expression profiles of individual integrant clones were compared within polyclonal populations using “zip coded” proviruses, each tagged to identify the genomic neighborhood where the provirus had integrated. The results revealed a complex array of heritable differences among clones in population sizes and expression characteristics.

Marking libraries with randomized sequence tags has been used in many systems including SIV and HIV-1 [39] [40] [16]. One group reported infectious SIV derivatives barcoded to track population dynamics during treatment and rebound [40]. Unlike those SIV derivatives [40], our vectors lacked Env and (except when remobilized by pseudotyping) were limited to single replication cycles. Barcodes were inserted toward the center of the virus in the SIV work, while ours were inserted near provirus edges to facilitate integration site determination. Another group described barcoded HIV-derived vectors called B-HIVE, with barcodes inserted in HIV-1’s multifunctional 5’ untranslated region. [16]. We chose to leave the 5’ leader region intact because it modulates HIV-1 expression by specifying nucleosome and transcription factor binding [9], folds into a finely-balanced equilibrium of RNA elements that regulate RNA fates [41], and is highly sensitive to mutation [42]. B-HIVE vectors encode LTR-driven GFP but no virus structural proteins. In contrast, our vectors retained *gag* and *pol*, thus allowing progeny virus production and the tracking of both virions and cellular nucleic acids. B-HIVE experiments were performed at a multiplicity of infection of 0.5 and likely included dually infected clones, while we used a much lower MOI. Additionally, we assessed expression in both unsorted cell pools and in serial sub-pools sorted for LTR reporter expression, and observed both dynamic and heritable aspects of clone-specific expression not evident in the B-HIVE work [16].

We benchmarked our system using a small (74 clones) pilot study that addressed replication fidelity. Zip code abundance varied widely in this pool, as did virus release per cell. Zip code survival rates suggested a single replication cycle lethal mutation rate in transformed cells of about 10%, but the true rate was likely lower than 10% because our vectors included non-viral sequences and the assay design introduced transmission bottlenecks. Most zip codes lost during the second cycle of replication were significantly less abundant in the initially infected cell pool than were those that persisted for the second round of replication, suggesting that population bottlenecks contributed to zip code extinction. Thus, in contrast to observations that the majority of patients’ persistent proviruses are defective even when sampled less than 60 days after initial infection [26, 28], the rate of mutational inactivation observed here was in the range predicted by previous work that suggests roughly one in three HIV-1 genomes accumulates any sort of reverse transcriptase-generated mutation per cycle of replication [43]. Thus, the difference between the relatively low rate of mutational inactivation here and the high prevalence of defective proviruses *in vivo* is consistent with the notion that the proviral landscape *in vivo* reflects selective pressures more than reverse transcriptase infidelity [28, 44].

Subsequent experiments were performed in Jurkat cells, using larger zip code libraries and proviruses with GFP in the *nef* open reading frame. In these experiments, we relied on GFP expression as a reporter of LTR activity, and did not assess expression by other means such as quantifying intracellular HIV-1 RNA or measuring virion release. HIV-1 proteins including Vpr and Env, which kill or inhibit cultured cells, were absent by design [45] [46]. Within the unsorted polyclonal Jurkat pool, GFP+ cells were more numerous than GFP- cells and virion release remained robust. As previously demonstrated with similar vectors, populations were readily separable into GFP+ and GFP- pools [47–49]. GFP+ pools displayed high levels of virion release while there was a near-absence of virus from GFP- cells. All abundant zip codes were reproducibly present in both GFP+ and GFP- cell sub-pools, but to widely varying extents. Using “GFP+ proportion” to represent the fraction of each clone’s cells that sorted GFP+, most clones were either “mostly GFP-” (with GFP+ proportions ≤0.4) or “mostly GFP+” (≥0.6). Although most cells in the unsorted pool were GFP+, the average number of cells per mostly GFP- Jurkat clone was significantly greater than for mostly GFP+ clones. This suggests that caution is appropriate when interpreting findings based on latency models that use GFP reporters and that passage cells until GFP activity largely disappears. Specifically, our results suggest that some of the apparent increases in latency over time may reflect outgrowth by clones with low GFP+ proportions rather than proviral silencing [50].

The stability of GFP+ proportions over time was addressed by re-sorting separately passaged GFP+ and GFP- sub-pools. Daughter cells did not always adopt a parental phenotype, but instead “flickered” between GFP+ and GFP-. When overall GFP+ values from the secondary sorts were compared to those from the sort 1 time point, the GFP+ proportions for each clone were remarkably similar over time. It is unclear whether the flickering observed here differs from the intraclonal expression variegation described previously within individual retroviral vector cell clones, which was interpreted to indicate integration site-dependent differences in silencing rather than alternating waves of expression [13, 51].

Heritable high levels of variation among HIV-1 integrant clones have been reported previously. However, unlike the flickering we observed, within-clone HIV-1 expression level variation has appeared relatively narrow using previous approaches [13, 51]. For example, wide inter-clone variation was reported in the B-HIVE study, but HIV-1 expression was quantified as intracellular RNA copies per cell barcode using an unsorted cell pool, and it was assumed that every cell within a given clone expressed LTR-driven RNAs to the same extent [16]. In contrast, because we determined that GFP positivity and intracellular p24 co-occurred in the polyclonal population, but that clones differed widely in their GFP+ proportions, our results suggest that at least part of the expression differences among clones reflects that each clone consists of a phenotypic mixture of cells—some that release virus and others that do not—in heritable clone-specific proportions.

What is responsible for these clone-specific stable equilibrium mixtures of GFP+ and GFP- cells? Intrinsic fluctuations in transcription factor availability and other stochastic events contribute significantly to gene expression, and can cause genetically identical cells propagated under uniform conditions to display a spectrum of phenotypes [52]. The sources, regulatory mechanisms, and implications of this genetic noise are active areas of investigation [53, 54]. Phenotypic bifurcation for HIV-1 infected cells, in which intrinsic noise in Tat expression leads to the co-existence within individual integrant clones of some cells that display high levels of expression and others that display essentially none, has previously been described [55]. Transcriptional bursting from the HIV-1 promoter is a significant source of stochastic noise [56], the bursting behavior of the export factor Rev may further exacerbate noise due to Tat [57], and the phase of the cell cycle may also exert influence [58]. These and other parameters likely contributed to the broad range of GFP+ proportion set points that differentiated clones here, even though our system was carried out in transformed cells with the intention of minimizing extrinsic variability [59].

The simplest explanation for why each clone adopted a unique GFP+ proportion set point may be that multiple inputs—some stochastic and others deterministic—combined in a clone-intrinsic manner to skew the probability that a given cell would reach the Tat threshold needed for GFP expression. The deterministic components could in concept be of either host or viral origin. However, our initial pilot experiment suggests that the principal differences were not within proviral sequences, but instead of host origin. Specifically, the amount of virus release per cell differed among zip codes when all cells with the same zip code were progeny of a single integration event, but virus release per cell was fairly uniform in a second generation when zip codes were polyclonal.

To explore host contributions due to integration site features, virus/host junctions were sequenced, integration sites determined, and the characteristics of mostly GFP+ and mostly GFP- clones compared. The results indicated that mostly GFP+ and mostly GFP- clones differed significantly in proviral orientation relative to host transcription. This may reflect transcriptional repression, which has been reported for HIV-1 [17, 60], although one study reported an opposite orientation bias [61]. We also assessed the correlations between repressive or activating chromatin marks previously determined in Jurkat cells [37, 38] and observed modest differences in proximity to H3K9me3 marks. Some previous studies appeared to find more conclusive correlates between epigenetic features and HIV expression [16] [17]. The less definitive trends reported here may be due to different approaches in measuring expression (RNA quantification vs. GFP+ proportions) or limited sample sets, and in some cases reflects how significance thresholds were defined. The magnitude of effects evident here suggests that our understanding of the roles of integration site features to robustly discriminate latency or viral expression remains incomplete.

Speculatively, some component of the observations here may reflect epigenetic marks introduced at the time of integration: due either to stochastic events or to differences in the intracellular environment or architecture of specific integration sites. It is generally assumed that most of the latent reservoir results from the rare infection of activated cells that transition to a memory state. However, HIV-1 can enter cells at any phase of the cell cycle. Histone biogenesis is cell cycle dependent [62] and many histone post-translational modifications are faithfully introduced onto nascent strands at the time of DNA replication. Although all epigenetic marks appear regenerated within the course of a single cell generation, some marks are copied with the replication fork while others (including H3K9me3 and H3K27me3) are deposited throughout the cell cycle [63, 64]. Because HIV can infect dividing or resting T cells, and the cell’s chromatin modification machinery displays cell cycle-dependent regulation, it is possible that integration at differing phases of the cell cycle results in distinct patterns of chromatin decoration [62, 65, 66].

It seems plausible that the HIV-1 expression variation reported here may cause some of the differences among experimental models for latency [18] and that expression flickering and differential set points of expression may be a fairly common outcome during the establishment of polyclonal HIV-1 populations. As such, these properties may contribute to defining the nascent proviral populations within infected people that are subsequently culled by immune and other selective pressures. Understanding how patterns of expression that persist compare to the palette of outcomes in the absence of selection may aid efforts to identify HIV-1’s epigenetic havens, and to the design of fruitful strategies for proviral eradication.

## Materials and methods

### Ethics statement

Peripheral blood mononuclear cells (PBMCs) were isolated from fresh human blood from healthy donors provided by the Department of Pathology at the University of Michigan. All samples were anonymized and all use of human samples was approved by the Institutional Review Board at the University of Michigan.

### Cell line propagation

293T cells were grown from a master cell bank [67] and Jurkat (Clone E6-1) cells were obtained from ATCC. Both cell lines were maintained as lab frozen stocks and validated at the time of study by tandem repeat analysis using the Applied Biosystems AmpFLSTR^™^ Identifiler^™^ Plus PCR Amplification Kit (Thermo Fisher Scientific, Carlsbad, CA). Jurkat cells were cultured in RPMI supplemented with 10% FBS (Gemini), 100 U/mL penicillin, 100 μg/mL streptomycin, 2mM glutamine and 55μM β-mercaptoethanol at 1 x 10^6^ cell/ml, while Human Embryonic Kidney (HEK) 293 T cells were grown in DMEM supplemented with 10% FBS (Gemini) and 125 μM gentamycin. Both cell lines were maintained in a 37°C incubator containing 5% CO_2_.

### Construction of zip coded vectors

All HIV-1 vectors were templated by derivatives of the NL4-3 strain plasmid NL4-3 GPP [30] or by HIV-GPV^-^, which was derived from the GKO [25] provided by Emilie Battivelli and Eric Verdin (University of California San Francisco). HIV-GPV^-^ was constructed by replacing mKO2 in GKO with the puromycin resistance gene from NL4-3 GPP. After initial work with standard two-LTR vectors, including the pilot fidelity study described here, subsequent zip coded vector preparation used single LTR versions of these vectors. For this, both vectors were modified into single “inside out” LTR forms containing the 5’ terminal 49 bases of U3 with an engineered Cla I site plus a second unique site (either Xho I or Mlu I) in U3, and inserted into pBR322 as previously described [68]. To generate zip coded HIV-1 vector templates, the single LTR plasmid versions of NL4-3 GPP and GPV- were digested with ClaI plus Xho I or Mlu I, respectively. The resulting 11.4kb HIV vector-containing fragments free of plasmid backbone were purified from agarose using QIAquick Gel Extraction Kit (Cat No./ID: 28706 Qiagen, Germantown, MD). A 304 bp zip code-containing insert fragment pool was generated by PCR using NL4-3 GPP or GPV^-^ as template, Phusion^®^ High-Fidelity DNA Polymerase (New England Biolabs, Inc., Ipswich, MA), and primers 5’-GACAAGATATCCTTGATCTGNNNNNNNNNNNNNNNNNNNNGCCATCGATGTGGATCTACCACACACAAGGC-3’ and 5’- CGGTGCCTGATTAATTAAACGCGTGCTCGAGACCTGGAAAAAC-3’ for GPV^-^ and 5’ GTGTGGTAGATCCACATCGATGGCNNNNNNNNNNNNNNNNNNNNCAGATCAAGGATATCTTGTCTTC-3’ and 5’- ATG CCA CGT AAG CGA AAC TCT CTG GAA GGG CTA ATT CAC TCC-3’ for NL4-3 GPP.

To generate the uncloned vector template library, the 11.4 kb fragments of GPV- or HIV-GPP were joined with their cognate 304 bp zip coded partial U3 inserts via Gibson Assembly in a molar ratio of 1:5 per reaction using HiFi DNA assembly mix (New England Biolabs) following the manufacturer’s protocol. The assembled DNA was then cleaned and concentrated using Zymo Clean and Concentrator-5 kit (SKU D4013 Zymo Research, Irvine, CA), quantified by Nanodrop (Thermo Fisher Scientific), and used directly in transfections.

### Virion production

Fresh monolayers of HEK 293T cells, in 10 cm diameter plates and approximately 70% confluent, were co-transfected with 3 μg Gibson Assembly product DNA plus 330 ng pHEF-VSV-G using polyethylenimine (Polysciences, Inc., Warrington, PA) at a ratio of 1 μg total DNA to 4 μg polyethylenimine in 800 μl of 150 mM NaCl [69]. 24 hours post-transfection, DMEM was replaced with 4 ml RPMI1640 medium with 10% FBS and 1% Pen/strep. Culture supernatant was harvested at 48 hours post-transfection and filtered through a 0.22 μm filter (Fisher Scientific. Cat. No. 09-720-511). Released virus was quantified using a real-time reverse-transcription PCR assay and normalized for p24 level based on p24 protein values determined in parallel for reference samples [68]. Zip coded virus stocks were titered by infecting 90% confluent HEK 293 T cells and selecting in puromycin. Colony forming units per milliliter of viral media as determined on 293T cells was the standard for defining infectious titer in this work.

### Infection of HEK 293 T and Jurkat cells

The media on 10 cm plates of 90% confluent HEK 293 T cells was replaced with 2000 μl infection mix comprised of the indicated amount of virus-containing medium plus additional DMEM in 1 μg/ml polybrene, then incubated at 37 °C with 5% CO_2_ for 5 hours. After incubation, the infection mix was replaced with 10 ml of fresh media. Twenty-four hours post-infection, cells were placed in media containing puromycin at a concentration of 1 μg/ml, which was replaced every three days for 2 weeks. Following this, colonies were individually cloned, pooled together for subsequent experiments, or stained with crystal violet and counted.

For Jurkat cell infections, virus-containing media and polybrene at a final concentration of 0.5 μg/ml were brought to a total volume of 1000 μl. This infection mixture was added to 1.5 x 10^6^ Jurkat cells and incubated in one well of a 12 well tissue culture plate (Fisher Scientific, Cat. 150628) at 37 °C with 5% CO_2_ for 5 hours. Infected cells were then transferred to Eppendorf tubes and centrifuged for 5 minutes at 2500 rpm at 4°C. Following centrifugation, supernatants were replaced with fresh media and cell pellets were resuspended and cultured at 37 °C with 5% CO_2_. At 24 hours post-infection, puromycin was added to a final concentration of 0.5 μg/ml. The infected cells were expanded into 6 cm culture plates without puromycin on day 5. Ten days post-infection, the culture supernatant was replaced with fresh media and the cultures were divided into aliquots, to be either frozen or further expanded for subsequent experiments.

### Primary T cell isolation and infection

Peripheral blood mononuclear cells (PBMCs) were isolated from fresh human blood from healthy donors provided by the Department of Pathology at the University of Michigan using Ficoll Histopaque as described earlier [70]. All use of human samples was approved by the Institutional Review Board at the University of Michigan. Total CD4+ T cells were then purified from PBMCs using MACS beads (Miltenyi Biotec Bergisch Gladbach, Germany) as per the manufacturer’s instructions. On day 0, a total of 5 x 10^6^ cells was seeded in complete culture medium composed of RPMI supplemented with 10% FBS, 100 U/mL penicillin, 100 μg/mL streptomycin, 2 mM glutamine and 55 μM β-mercaptoethanol at 1 x 10^6^ cell/ml. The cells were stimulated using plate-bound anti-CD3 (5 μg/mL; eBioscience, Thermo Fisher Scientific) and soluble anti-CD28 (1 μg/mL; eBioscience, Thermo Fisher Scientific) antibodies in the presence of 50 U/ml IL-2 (PeproTech, Inc., Rocky Hill, NJ). On day 2 of activation, the cells were infected by spinoculation at 2500 rpm for 90 minutes at 37° C with 125 μL zip coded viral media and 0.4 μg/ml polybrene (Sigma Aldrich, St. Louis, MO) in 2.5 ml of supplemented RPMI. After spinoculation, media containing virus was replaced with fresh supplemented RPMI and cells were cultured further and expanded as needed. On day 6 or 7 post-activation, cells were harvested and sorted into GFP^+^ and GFP^-^ sub-pools by flow cytometry using FACS Aria II (BD Biosciences, Franklin Lakes, NJ) or iCyt Synergy SY3200 (Sony Biotechnology, San Jose, CA) cell sorter. Selective drugs were not applied in these primary cell experiments. Thus, the GFP- sub-pools included uninfected cells, and the fraction of infected primary cells that were GFP+ was not experimentally determined. A 5% GFP+ value was selected for use as an assumed value in the comparison of primary cell clones, based on observations that 10% GFP+ is on the upper edge of previously reported values for primary cells infected with similar vectors, with values less than 3% more typical, possibly reflecting donor-dependent variation or survival of some non-transduced cells [71]. Thus a 5% value was used as a conservative measure, to spread data points that would have appeared similar if a value <3% were used. Importantly, note that although absolute values would change if true GFP- value were higher or lower than this assumed value, correlation values and their interpretation would not be affected.

### Flow cytometry

For flow cytometry analysis and sorting, Jurkat or primary T cells were suspended in phosphate buffered saline (PBS) containing 1% FBS (FACS buffer). Dead cells were excluded in all analyses and sorting experiments using propidium iodide (PI). Intracellular Gag staining was carried out using a Gag monoclonal antibody conjugated to Phycoerythrin (KC-57 RD1 Beckman Coulter). 1x10^5^ cells from a HIV GPV- zip coded library were washed once with FACS buffer and fixed with 100 μl of BD cytofix for 10 minutes at room temperature in the dark. Cells were then washed twice with FACS buffer then once with BD perm/wash buffer. Staining was carried out at a 1:200 dilution of antibody in 1x BD perm buffer. The cells were incubated in the dark at room temperature for 15 minutes, washed twice, then resuspended in 200 μl FACS buffer. Acquisition was carried out on the FITC channel for GFP and PE channel for Gag. Cell fluorescence was assessed using FACSCanto II (BD Biosciences) and data were analyzed using FlowJo software, version 9.9 (FlowJo, LLC., Ashland, Oregon).

### PCR amplification of zip codes from zip coded cells and virus

Genomic DNA was extracted from zip coded cell libraries using Qiagen DNeasy Blood & Tissue Kit (Qiagen, Germantown, MD). Zip codes were amplified from 100 ng of genomic DNA using primers flanking the zip code region (primers: 5‘-NNACGAAGACAAGATATCCTTGATC-3’ and 5’-NNTGTGTGGTAGATCCACATCG-3’) using Phusion^®^ High-Fidelity DNA Polymerase (New England Biolabs) in HF Buffer. For zip code amplification, we designed multiple primers complementary to the template binding site that included two known, random nucleotides at the 5’ end for use in separate reactions. By comparing the primers used for amplification and the nucleotides at the end of each amplicon, we could confirm that PCR cross contamination had not occurred. Reactions were cycled 26–35 times with 30 second extension at 72° and a 59° annealing temperature. Zip coded amplicons were purified with DNA Clean & Concentrator-5 (Zymo Research, CA. Cat. No. D4013) and eluted in 20 μl of H_2_O. To amplify zip codes from virus, virus-containing media was filtered through a 0.22 μm filter, concentrated by ultracentrifugation at 25,000 rpm through a 20% sucrose cushion, and RNA extracted with Invitrogen TRIzol Reagent (Thermo Fisher Scientific). The dissolved RNA was treated with RQ1 DNase (Promega, Fitchburg, WI) to remove possible DNA traces, re-extracted with phenol-chloroform, and stored at −80° C. cDNA was synthesized using M-MLV RT (H–) (Promega) and U3 antisense primer 5’-TGTGTGGTAGATCCACATCG-3’. Zip codes were amplified from this cDNA using conditions outlined above.

For library construction, protocols and reagents from NEBNext^®^ Ultra^™^ DNA Library Prep Kit for Illumina^®^ (New England Biolabs) were used for end repair, dA-tailing, and to ligate Nextflex adapters (Perkin Elmer, Waltham, MA) onto amplicons. After ligation, reactions were diluted up to 100 μl with H_2_O, purified with 0.85x SPRIselect beads, washed twice in 70% ethanol, and eluted into H_2_O. PCR enrichment of adapter-ligated amplicons was done for 7 cycles using NEBNext^®^ Ultra^™^ DNA Library Prep Kit, reactions were diluted up to 100 μl with H_2_O, and purified with 0.85x SPRIselect beads (Beckman Coulter) as outlined above. Libraries were quantitated with KAPA Library Quantification Kits for Next-Generation Sequencing (Roche Sequencing Solutions, Inc., Pleasanton, CA) and Qubit^™^ dsDNA HS Assay Kit (Thermo Fisher Scientific), pooled equally, and sequenced with MiSeq Reagent Kit v3, 150 cycle PE on a MiSeq sequencer (Illumina, San Diego, CA).

### Calculating GFP+ proportions

GFP+ proportions were calculated by dividing the read frequency of each zip code within GFP+ sorted cells by the summed abundance of the zip code in both GFP+ and GFP- sorted cells, after weighting values to reflect the fractions of total cells that sorted into GFP+ and GFP- sub-pools. For example, GFP+ read frequency of a clone would be the proportion of GFP+ total reads that contained that zip code. If the total pool was 75% GFP+ and 25% GFP- cells and a given zip code were 2% of the GFP+ cells and 3% of the GFP- cells, the GFP+ proportion of that clone would be (2% of 75%) / (2% of 75% + 3% of 25%) = 67%.

### HIV integration-site sequencing

Template for hemi-specific ligation mediated PCR of insertion sites was obtained by linear PCR and biotin enrichment of sheared, genomic DNA with linkers ligated on each end. Linker was synthesized by mixing oligo 5'–GTAATACGACTCACTATAGGGCTCCGCTTAAGGGACT-3’ and 5'–PO4- GTCCCTTAAGCGGAG-3’-C6 [72] at a final concentration of 40 μM each in 100 μl volume. Oligo mixture was heated in a PCR block for 5 minutes at 95°C, the PCR machine was immediately shut off, and the block was allowed to cool for 2 hours to room temperature. Genomic DNA was extracted from cells using Qiagen DNeasy Blood & Tissue kit (Qiagen) and 200 ng of DNA was sheared to 1 kb fragments using Covaris M220 and micro-TUBE according to the manufacturer’s recommended settings (Covaris, Woburn, MA). Sheared DNA was purified with 1x SPRIselect beads according to the manufacturer’s instructions (Beckman Coulter) and sheared ends were repaired with NEBNext^®^ Ultra^™^ End Repair/dA-Tailing Module (New England Biolabs) using the manufacturer’s protocol. Repaired, dA-tailed DNA was purified with 0.7x SPRIselect beads (Beckman Coulter) and the partially double stranded DNA linker with dT overhang was ligated in a 60 μl reaction containing 6 μl of 10X T4 DNA Ligase Buffer, 1.33 μM linker DNA, and 3600 U Ultrapure T4 DNA ligase (Qiagen) at 16° C for 16 hours followed by 70° C incubation for 10 minutes. Ligated DNA was purified with 0.7 x SPRIselect beads (Beckman Coulter) and used for template in a linear PCR reaction containing 1x Expand Long Range Buffer, 500 μM dNTPs, 3% DMSO, 3.5U Long Range Enzyme Mix, and a 500 μM biotinylated primer that anneals to the HIV LTR in our construct, 5’- /52-Bio/CAAAGGTCAGTGGATATCTGACCCC-3’. Cycling parameters were 95° C for 5 minutes, 40 cycles of 95° C for 45 seconds, 60° C for 1 minute, and 68° C for 1.5 minutes, followed by a 10 minute incubation at 68° C. PCR product was purified with 1x SPRIselect beads (Beckman Coulter), resuspended in 20 μl H_2_O, and biotinylated fragments were captured using Dynabeads kilobase BINDER kit (Thermo Fisher Scientific) according to the manufacturer’s instructions. DNA captured by beads was used as template in a hemi-specific PCR reaction containing 1x Expand Long Range Buffer, 500 μM dNTPs, 3% DMSO, 3.5 U Long Range Enzyme Mix, 500 μM of a nested primer that anneals to the HIV LTR in our construct, 5’-GCCAATCAGGGAAGTAGCCTTGTGTGTGG-3’, and 500 μM of a primer that anneals to the linker, 5’-AGGGCTCCGCTTAAGGGAC-3’. Cycling parameters were 95° C for 5 minutes, 30 cycles of 95° C for 45 seconds, 60° C for 1 minute, and 68° C for 1.5 minutes, followed by 10 minutes’ incubation at 68° C. PCR product was purified with 0.7x SPRIselect beads (Beckman Coulter), then protocol and reagents from NEBNext^®^ Ultra^™^ DNA Library Prep Kit for Illumina (New England Biolabs) were used to end repair, dA-tail, and ligate Nextflex sequencing adapters (Perkin Elmer) onto amplicons. Ligation reaction was purified with 0.65x SPRIselect beads (Beckman Coulter), and 7 cycles of PCR to enrich for ligated product was done with NEBNext^®^ Ultra^™^ DNA Library Prep Kit for Illumina (New England Biolabs). Libraries were quantitated with KAPA Library Quantification Kits for Next-Generation Sequencing (Roche Sequencing Solutions, Inc., Pleasanton, CA) and Qubit^™^ dsDNA HS Assay Kit (Thermo Fisher Scientific), pooled equally, and sequenced with MiSeq Reagent Kit v3, 600 cycle PE on MiSeq sequencer (Illumina, San Diego, CA). All generated sequence data has been deposited to the Sequence Read Archive (SRA) under project accession PRJNA531502.

### Zip code analysis and quantification

Zip codes were identified and quantified from Illumina sequencing reads using a custom suite of tools implemented in Python (https://github.com/KiddLab/hiv-zipcode-tools). First, 2x75 bp paired reads were merged together using *flash* v1.2.11 [73]. Zip codes were identified by searching for known flanking sequence (with up to 1 mismatch). Only candidate zip codes with a length of 17–23 nucleotides were considered and the read count for each unique zip code was tabulated. To identify the set of zip codes for further analysis, zip code families which account for PCR and sequencing errors were determined by clustering together the observed unique zip codes. Comparisons among zip codes were calculated using a full Needleman-Wunch alignment tabulated with a score of +1 for sequence matches, -1 for mismatches, and a constant gap score of -1. Comparisons with two or fewer mismatches (counting a gap as a mismatch) were accepted as a match. Using this criteria clusters were then identified. First, unique zip codes were sorted by abundance. Then, beginning with the most abundant zip code, each sequence was compared with all of the previous zip codes. If no previous zip code had two or fewer mismatches that zip code was accepted as a cluster and then the next most abundant zip code was considered. This process was continued until the first unique zip code having a match to a more abundant zip code was identified. This defined the set of families for consideration. Abundance for the families was then determined by assigning unique zip codes to the most abundant family whose sequence was within 2 mismatches and summing their associated read counts.

In sorting experiments, the GFP+ proportion for each zip code was determined as F_i_ = (G_i_ * P)/ (G_i_ * P + W_i_ * Q) where F_i_ is the GFP+ fraction of zip code i, G_i_ is the fraction abundance of zip code i in the GFP+ sorted pool, W_i_ is the fraction abundance of zip code i in the GFP- sorted pool, P is the fraction of cells that sorted into the GFP+ pool and Q is the fraction of cells that sorted into the GFP- pool. In the Jurkat pool 1, the initial GFP+ fraction was 0.524 and the initial GFP- fraction was 0.36. Of the GFP+ sort from pool 1 the GFP+ fraction was 0.887 and the GFP- fraction was 0.079 GFP- while in the GFP- sort from pool 1 the GFP+ fraction was 0.046 and the GFP- fraction was 0.928. In the Jurkat pool 2, the initial GFP+ fraction was 0.518 and the initial GFP- fraction was 0.364. Of the GFP+ sort from pool 2 the GFP+ fraction was 0.915 and the GFP- fraction was 0.082 GFP- while in the GFP- sort from pool 2 the GFP+ fraction was 0.063 and the GFP- fraction was 0.923. For primary cell data analysis, the abundance of each zip codes in the GFP+ and GFP- pools summed, and only those zip codes with summed abundance greater than 0.0001 in both replicates were considered, and a GFP+ fraction of 0.95 and a GFP- fraction of 0.05 were assumed.

Analysis of integration sites occurred in two stages. First, read-pairs were analyzed to identify which read derived from the LTR sequence and which from the genomic linker. Zip code sequences were extracted from the LTR-derived read based on matches to flanking sequence in the vector as described above. The linker sequence and LTR sequence flanking the zip code were removed and the extracted zip code sequence was then associated with the remaining portion of each read pair. Second, the trimmed read pairs were aligned to a version of the hg19 genome that included the sequence of the utilized HIV vector using bwa mem version 0.7.15. The resulting alignments were then parsed to identify the shear point (DNA adjacent to where the linker was ligated) and integration point (the DNA location adjacent to the LTR sequence). The zip codes were then assigned to previously identified zip code families, and the number of unique shear points and total reads supporting a integration site for each zip code were tabulated. Only reads with a mapping quality greater than 10 were considered, and sonication breakpoints that appear within 3 nucleotides of one another were considered to represent the same shear point [72]. A greedy algorithm was then used to associate each zip code with a genomic location, to remove minor assignments presumed to be chimeras generated as PCR artifacts. “Greedy strategy” is a term from computer science that refers to an algorithm which solves a multi-part problem by dividing the problem into separate states or pieces and then selecting the outcome that maximizes an indicated criteria at each stage [74]. We assigned zip codes to genome locations based on the number of supporting fragments. First, we assign the zip code with the largest number of fragments to the location supported by the most fragments. Next, other fragments associated with that zip code are removed from consideration. This process is then repeated for the remaining zip code with the largest number of supporting fragments.

### Determination of chromatin marks and expressed genes

Gene annotations were determined based on Ensembl release 75. Jurkat gene expression data produced by Encode [75] was used (accession ENCSR000BXX), and genes with TPM counts greater than 5 in both replicated were considered to be expressed. H3K27ac peaks were identified using data from [37] (GSM1697880 and GSM1697882). Chip-seq and control data were aligned to hg19 using bwa mem and peaks were identified using macs2 v 2.1.0 [76] with the—nomodel option. For H3K9me3 peaks, data from [38] (GSM1603227) were aligned to hg19 using bwa mem and processed using macs2 without a control sequence set. For both marks a p value cutoff of 1*10^−9^ was used.

## Supporting information

S1 Fig Zip code complexity in Gibson assembly mix used to generate zip coded virion RNAs. A zip code amplicon was made from 1% of the Gibson assembly mix used in transfections to generate zip coded virus. The amplicon was high throughput sequenced and zip codes were clustered into zip code families. Of 6.23 million sequencing reads, the plot shows ~4% of the reads (right axis: red) contained 100,000 zip codes (left axis: blue). Zip code rank refers to the order of zip codes, sorted by read abundance.
(PDF)

S2 Fig Zip code family and read abundance for single cycle pilot experiment. The red line (right axis) show the cumulative fraction of reads accounted for by each unique zip code. The blue line (left axis) shows the number of unique zip code families determined after clustering the indicated number of unique zip codes. The inflection point on the blue line indicated that the zip codes clustered into 74 families.
(PDF)

S3 Fig Analysis of Jurkat cell pool high throughput sequencing reads and assignment of zip code families. **Zip code fractional abundance**. Each of 706 zip code families identified in the Jurkat pool is depicted by a single point. The clones are arrayed left to right from the most abundant to the least abundant, with the fractional abundance of total reads assigned to that zip code on the Y axis. **Zip code rank and fractional abundance for Jurkat pool**. The red line (right axis) show the cumulative fraction of reads accounted for by each unique zip code. The blue line (left axis) shows the number of zip code families determined by clustering the indicated number of unique zip codes.
(PDF)

S4 Fig Gating of GFP+ and GFP- subpopulations for sorting. Prior to sorting, cells were stained with propidium iodide. (A) Uninfected Jurkat cells were gated based on FSC-Area and SSC-A to gate out cellular debris (panel 1), followed by gates based on FSC and SSC widths and heights to exclude doublets (panels 2 and 3). Next, the propidium iodide positive cells were gated out using the PE channel to exclude dead cells (panel 4). Lastly, GFP- and GFP+ gates were drawn in the FITC channel as shown panel 5. These gates were then applied to (B) Pool 1, and (C) Pool 2 to sort GFP+ and GFP-.
(PDF)

S5 Fig Flow cytometric analysis for the co-occurrence of intracellular Gag staining and GFP. Performed using Jurkat cells containing zip coded HIV GPV- library as described in Materials and Methods. Numbers in each quadrant indicate the proportion of total cells in that quadrant.
(PDF)

S6 Fig GFP+ fractions in primary cells *Scatter plots of GFP+ proportions for three primary cell experiments*. The percent GFP+ for each zip code was calculated assuming 95% of cells were GFP+. Inclusion criteria were selected to identify zip codes sufficiently abundant following the limited cellular divisions in the passaged primary cells. For each zip code plotted, the abundance fraction determined in summed GFP+ and GFP- sorts was required to be >0.0001. **(A)** Scatter plot of GFP+ proportions for experiment 1. A total of 349 zip codes passed the inclusion criteria, and show a Spearman correlation of ρ = 0.367 (p = 6.45x10^-12^) among SplitA and SplitB replicates. **(B)** GFP+ proportions from experiment 2. 73 zip codes passed the inclusion criteria, with a Spearman correlation of ρ = 0.719 (p = 7.65x10^-13^). **(C)** GFP+ proportions from experiment 3. 90 zip codes passed the inclusion criteria, with a Spearman correlation of ρ = 0.730 (p = 3.22x10^-16^). In each case, points are colored based on average fraction abundance in the GFP+ pools (green color bar). We note that sequencing libraries for experiments 2 and 3 were prepared at the same time and 6 additional PCR cycles were required due to low input of starting material. Some high abundance zip codes, representing potential contamination, were found in both experiment 2 and 3 libraries and were removed from all analyses. Note also that in primary cell experiments, many zip codes were detected in only one pool, likely representing unintegrated viral DNA or infected cells that had divided too few times to be sampled evenly. For example, aliquots of experiments 2 and 3 unsorted infected cell pools displayed totals of 43,525 and 33,114 zip codes, respectively. Of these, 35,686 (82%) or 28,842 (87%) were not observed in either GFP+ or GFP- fractions of SplitA or SplitB. This high rate of zip codes observed only in the unsorted pool illustrates the challenges in the primary cell analysis, which relies upon sufficient cellular divisions for zip codes to be detected in a reproducible manner among split pools.
(PDF)

S1 Table Randomized sequence tags in trial proviral clones. (PDF)

S2 Table Integration sites. (PDF)
