# Supplementary material for: Stable integrant-specific differences in bimodal HIV-1 expression patterns revealed by high-throughput analysis
Source: PLoS Pathog. 2019 Oct 4;15(10):e1007903. doi: 10.1371/journal.ppat.1007903 (PMC6795456; doi:10.1371/journal.ppat.1007903)
Supplement: S1 Fig — A zip code amplicon was made from 1% of the Gibson assembly mix used in transfections to generate zip coded virus. The amplicon was high throughput sequenced and zip codes were clustered into zip code families. Of 6.23 million sequencing reads, the plot shows ~4% of the reads (right axis: red) contained 100,000 zip codes (left axis: blue). Zip code rank refers to the order of zip codes, sorted by read abundance. (PDF) [file ppat.1007903.s001.pdf]

S1 Fig: **Zip code complexity in Gibson assembly mix used to generate zip coded virion RNAs**

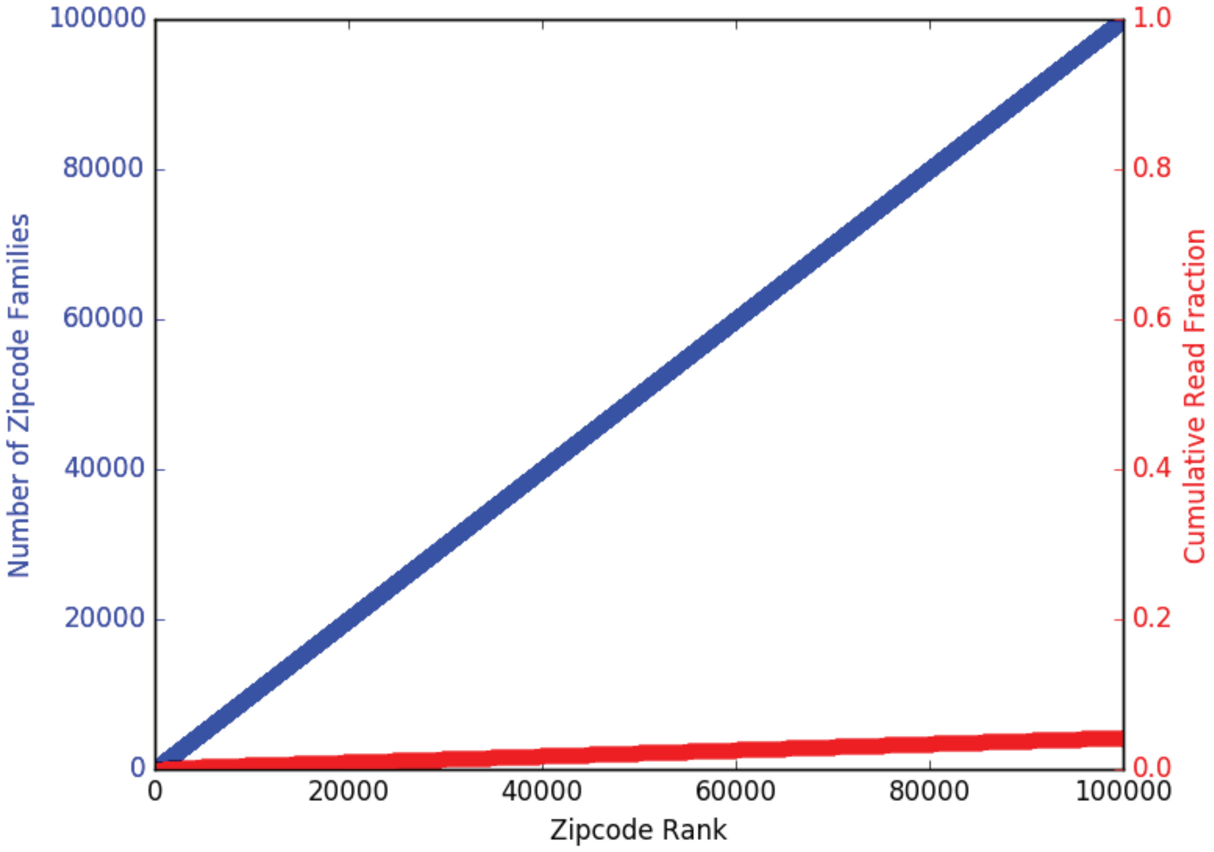

A zip code amplicon was made from 1% of the Gibson assembly mix used in transfections to generate zip coded virus. The amplicon was high throughput sequenced and zip codes were clustered into zip code families. Of 6.23 million sequencing reads, the plot shows ~4% of the reads (right axis: red) contained 100,000 zip codes (left axis: blue). Zip code rank refers to the order of zip codes, sorted by read abundance.
