# Supplementary material for: Stable integrant-specific differences in bimodal HIV-1 expression patterns revealed by high-throughput analysis
Source: PLoS Pathog. 2019 Oct 4;15(10):e1007903. doi: 10.1371/journal.ppat.1007903 (PMC6795456; doi:10.1371/journal.ppat.1007903)
Supplement: S2 Fig — The red line (right axis) show the cumulative fraction of reads accounted for by each unique zip code. The blue line (left axis) shows the number of unique zip code families determined after clustering the indicated number of unique zip codes. The inflection point on the blue line indicated that the zip codes clustered into 74 families. (PDF) [file ppat.1007903.s002.pdf]

S2 Fig: **Zip code family and read abundance for single cycle pilot experiment**

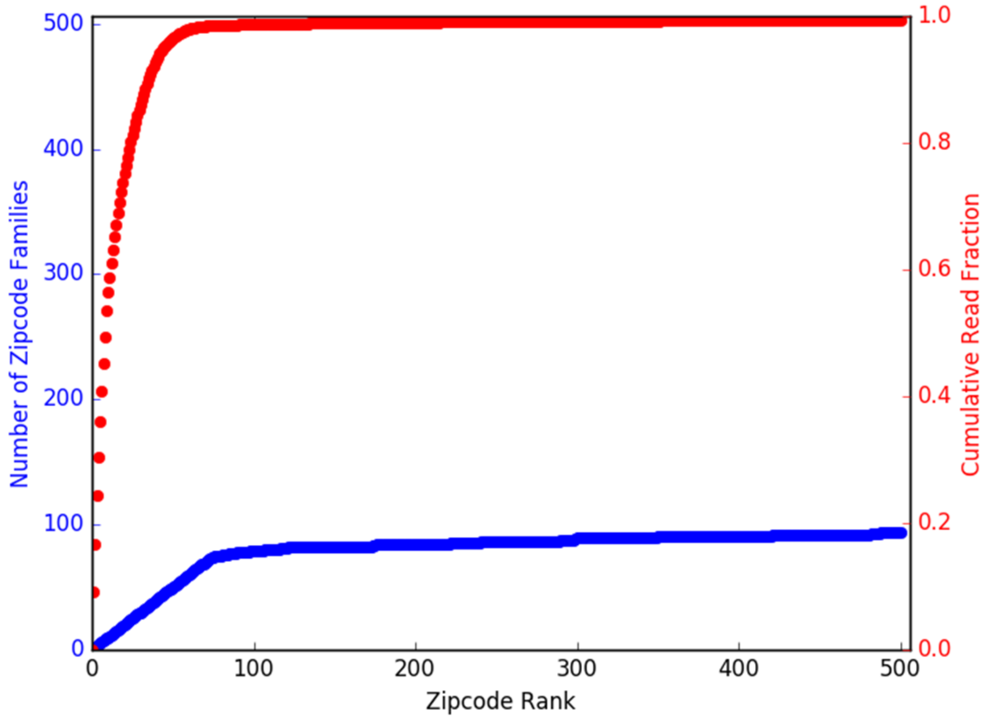

The red line (right axis) show the cumulative fraction of reads accounted for by each unique zip code. The blue line (left axis) shows the number of unique zip code families determined after clustering the indicated number of unique zip codes. The inflection point on the blue line indicated that the zip codes clustered into 74 families.
