# Supplementary material for: Stable integrant-specific differences in bimodal HIV-1 expression patterns revealed by high-throughput analysis
Source: PLoS Pathog. 2019 Oct 4;15(10):e1007903. doi: 10.1371/journal.ppat.1007903 (PMC6795456; doi:10.1371/journal.ppat.1007903)
Supplement: S3 Fig — Zip code fractional abundance. Each of 706 zip code families identified in the Jurkat pool is depicted by a single point. The clones are arrayed left to right from the most abundant to the least abundant, with the fractional abundance of total reads assigned to that zip code on the Y axis. Zip code rank and fractional abundance for Jurkat pool. The red line (right axis) show the cumulative fraction of reads accounted for by each unique zip code. The blue line (left axis) shows the number of zip code families determined by clustering the indicated number of unique zip codes. (PDF) [file ppat.1007903.s003.pdf]

S3 Fig: **Analysis of Jurkat cell pool high throughput sequencing reads and assignment of zip code families**

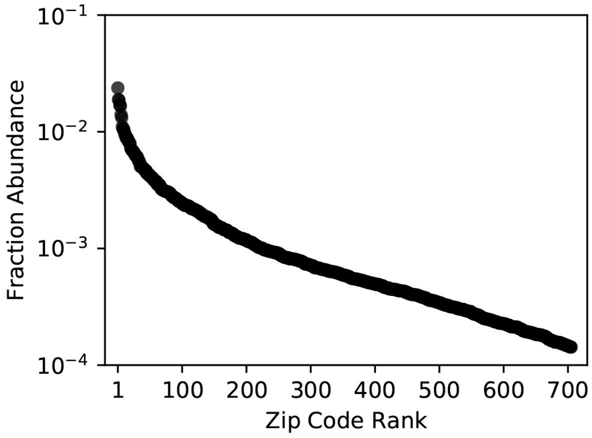

Zip code fractional abundance. Each of 706 zip code families identified in the Jurkat pool is depicted by a single point**.** The clones are arrayed left to right from the most abundant to the least abundant, with the fractional abundance of total reads assigned to that zip code on the Y axis.

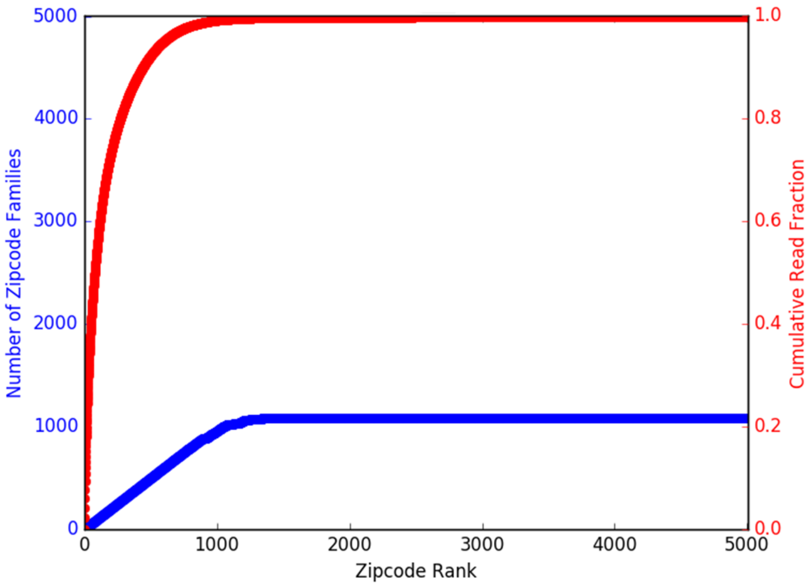

Zip code rank and fractional abundance for Jurkat pool. The red line (right axis) show the cumulative fraction of reads accounted for by each unique zip code. The blue line (left axis) shows the number of zip code families determined by clustering the indicated number of unique zip codes.
