# Supplementary material for: Stable integrant-specific differences in bimodal HIV-1 expression patterns revealed by high-throughput analysis
Source: PLoS Pathog. 2019 Oct 4;15(10):e1007903. doi: 10.1371/journal.ppat.1007903 (PMC6795456; doi:10.1371/journal.ppat.1007903)
Supplement: S4 Fig — Prior to sorting, cells were stained with propidium iodide. (A) Uninfected Jurkat cells were gated based on FSC-Area and SSC-A to gate out cellular debris (panel 1), followed by gates based on FSC and SSC widths and heights to exclude doublets (panels 2 and 3). Next, the propidium iodide positive cells were gated out using the PE channel to exclude dead cells (panel 4). Lastly, GFP- and GFP+ gates were drawn in the FITC channel as shown panel 5. These gates were then applied to (B) Pool 1, and (C) Pool 2 to sort GFP+ and GFP-. (PDF) [file ppat.1007903.s004.pdf]

S4 Fig: **Gating of GFP+ and GFP- subpopulations for sorting**

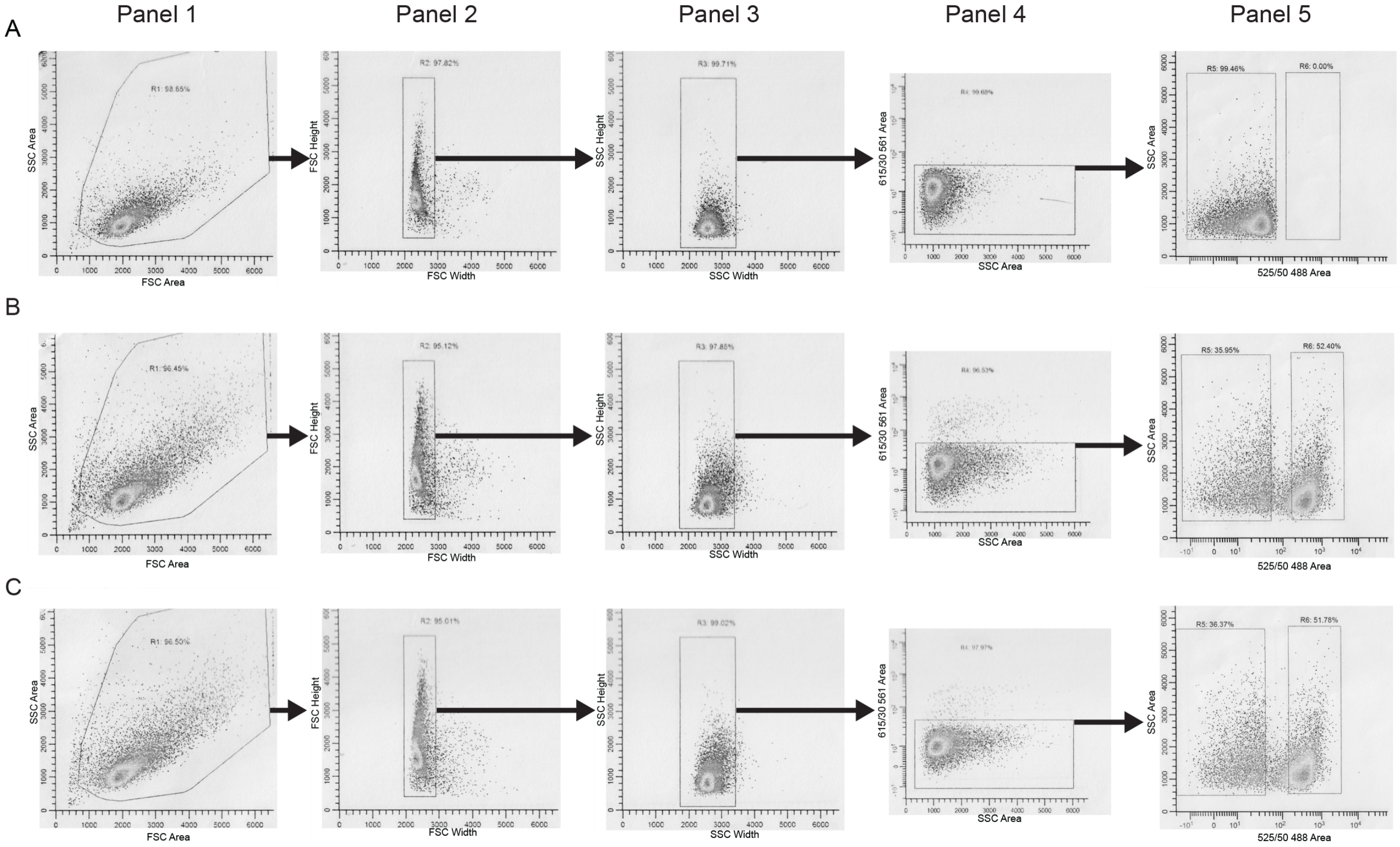

Prior to sorting, cells were stained with propidium iodide. (A) Uninfected Jurkat cells were gated based on FSC-Area and SSC-A to gate out cellular debris (panel 1), followed by gates based on FSC and SSC widths and heights to exclude doublets (panels 2 and 3). Next, the propidium iodide positive cells were gated out using the PE channel to exclude dead cells (panel 4). Lastly, GFP- and GFP+ gates were drawn in the FITC channel as shown panel 5. These gates were then applied to (B) Pool 1, and (C) Pool 2 to sort GFP+ and GFP-.
