# Supplementary material for: Stable integrant-specific differences in bimodal HIV-1 expression patterns revealed by high-throughput analysis
Source: PLoS Pathog. 2019 Oct 4;15(10):e1007903. doi: 10.1371/journal.ppat.1007903 (PMC6795456; doi:10.1371/journal.ppat.1007903)
Supplement: S5 Fig — Performed using Jurkat cells containing zip coded HIV GPV- library as described in Materials and Methods. Numbers in each quadrant indicate the proportion of total cells in that quadrant. (PDF) [file ppat.1007903.s005.pdf]

S5 Fig: **Flow cytometric analysis for the co-occurrence of intracellular Gag staining and GFP**

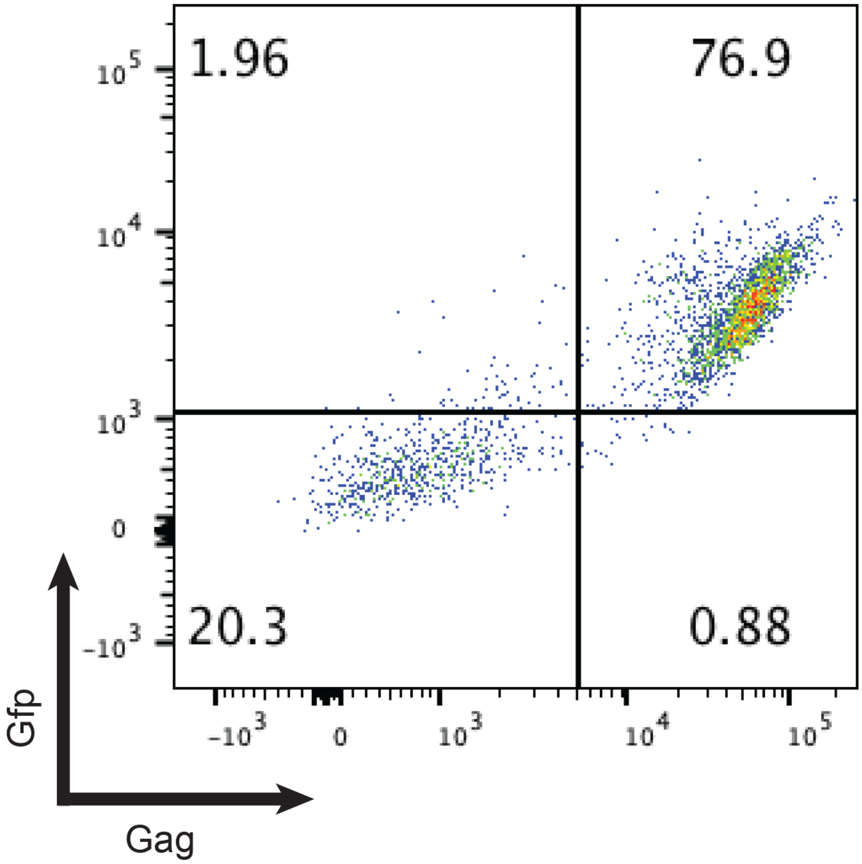

Performed using Jurkat cells containing zip coded HIV GPV- library as described in Materials and Methods. Numbers in each quadrant indicate the proportion of total cells in that quadrant.
