# Supplementary material for: Stable integrant-specific differences in bimodal HIV-1 expression patterns revealed by high-throughput analysis
Source: PLoS Pathog. 2019 Oct 4;15(10):e1007903. doi: 10.1371/journal.ppat.1007903 (PMC6795456; doi:10.1371/journal.ppat.1007903)
Supplement: S6 Fig — The percent GFP+ for each zip code was calculated assuming 95% of cells were GFP+. Inclusion criteria were selected to identify zip codes sufficiently abundant following the limited cellular divisions in the passaged primary cells. For each zip code plotted, the abundance fraction determined in summed GFP+ and GFP- sorts was required to be >0.0001. (A) Scatter plot of GFP+ proportions for experiment 1. A total of 349 zip codes passed the inclusion criteria, and show a Spearman correlation of ρ = 0.367 (p = 6.45x10^-12^) among SplitA and SplitB replicates. (B) GFP+ proportions from experiment 2. 73 zip codes passed the inclusion criteria, with a Spearman correlation of ρ = 0.719 (p = 7.65x10^-13^). (C) GFP+ proportions from experiment 3. 90 zip codes passed the inclusion criteria, with a Spearman correlation of ρ = 0.730 (p = 3.22x10^-16^). In each case, points are colored based on average fraction abundance in the GFP+ pools (green color bar). We note that sequencing libraries for experiments 2 and 3 were prepared at the same time and 6 additional PCR cycles were required due to low input of starting material. Some high abundance zip codes, representing potential contamination, were found in both experiment 2 and 3 libraries and were removed from all analyses. Note also that in primary cell experiments, many zip codes were detected in only one pool, likely representing unintegrated viral DNA or infected cells that had divided too few times to be sampled evenly. For example, aliquots of experiments 2 and 3 unsorted infected cell pools displayed totals of 43,525 and 33,114 zip codes, respectively. Of these, 35,686 (82%) or 28,842 (87%) were not observed in either GFP+ or GFP- fractions of SplitA or SplitB. This high rate of zip codes observed only in the unsorted pool illustrates the challenges in the primary cell analysis, which relies upon sufficient cellular divisions for zip codes to be detected in a reproducible manner among split pools. (PDF) [file ppat.1007903.s006.pdf]

S6 Fig: **GFP+ fractions in primary cells**

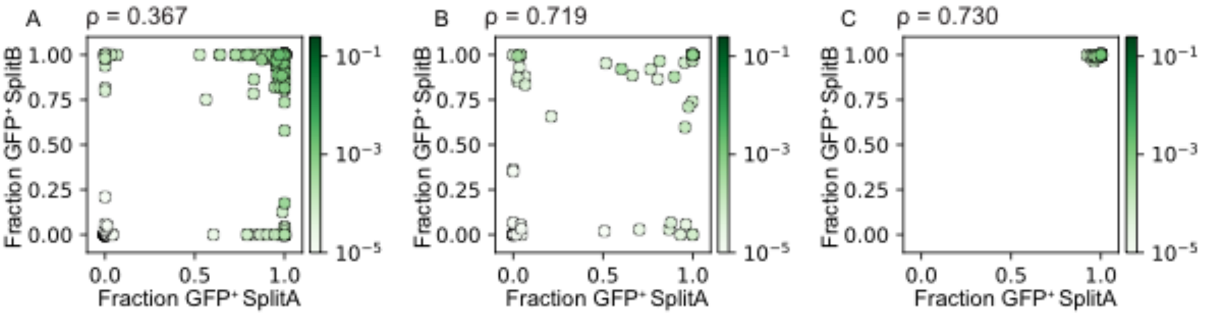

Scatter plots of GFP+ proportions for three primary cell experiments. The percent GFP+ for each zip code was calculated assuming 95% of cells were GFP+. Inclusion criteria were selected to identify zip codes sufficiently abundant following the limited cellular divisions in the passaged primary cells. For each zip code plotted, the abundance fraction determined in summed GFP+ and GFP- sorts was required to be >0.0001**. (A)** Scatter plot of GFP+ proportions for experiment 1. A total of 349 zip codes passed the inclusion criteria, and show a Spearman correlation of $\rho=0.367$ ($p=6.45 \times 10^{-12}$) among SplitA and SplitB replicates. **(B)** GFP+ proportions from experiment 2. 73 zip codes passed the inclusion criteria, with a Spearman correlation of $\rho=0.719$ ($p=7.65 \times 10^{-13}$). **(C)** GFP+ proportions from experiment 3. 90 zip codes passed the inclusion criteria, with a Spearman correlation of $\rho=0.730$ ($p=3.22 \times 10^{-16}$). In each case, points are colored based on average fraction abundance in the GFP+ pools (green color bar). We note that sequencing libraries for experiments 2 and 3 were prepared at the same time and 6 additional PCR cycles were required due to low input of starting material. Some high abundance zip codes, representing potential contamination, were found in both experiment 2 and 3 libraries and were removed from all analyses. Note also that in primary cell experiments, many zip codes were detected in only one pool, likely representing unintegrated viral DNA or infected cells that had divided too few times to be sampled evenly. For example, aliquots of experiments 2 and 3 unsorted infected cell pools displayed totals of 43,525 and 33,114 zip codes, respectively. Of these, 35,686 (82%) or 28,842 (87%) were not observed in either GFP+ or GFP- fractions of SplitA or SplitB. This high rate of zip codes observed only in the unsorted pool illustrates the challenges in the primary cell analysis, which relies upon sufficient cellular divisions for zip codes to be detected in a reproducible manner among split pools.

Experimental repetition	SplitA/SplitB %GFP+	# zip codes that pass inclusion criteria	Spearman correlations
A	0.4/0.41	349	0.367
B	0.2/0.22	73	0.719
C	1.0/0.9	90	0.730

Summarized data for the three primary cell experiments. Note that the GFP- sorted cells included uninfected cells. Experimental information is provided above and in Materials and Methods.
