# Supplementary material for: Stable integrant-specific differences in bimodal HIV-1 expression patterns revealed by high-throughput analysis
Source: PLoS Pathog. 2019 Oct 4;15(10):e1007903. doi: 10.1371/journal.ppat.1007903 (PMC6795456; doi:10.1371/journal.ppat.1007903)
Supplement: S1 Table — (PDF) [file ppat.1007903.s007.pdf]

S1 Table: **Randomized sequence tags in trial proviral clones**

| | |
|---|---|
| Library | gccatcgatgNNNNNNNNNNNNNNNNNNNNtggatctaccac |
| Clone 1 | gccatcgatgGGTACGGTGGTCATAGCATGtggatctaccac |
| Clone 2 | gccatcgatgGCTCGCGACTGGGGGTGGTGtggatctaccac |
| Clone 3 | gccatcgatgGGTTAGTTGGCGCAAGTGCTtggatctaccac |
| Clone 4 | gccatcgatgTGTCTCATCGGACGGAGGATtggatctaccac |
| Clone 5 | gccatcgatgACATCATGTGTCGCTCCTGCtggatctaccac |
| Clone 6 | gccatcgatgAGCTCGGAGCGTGCGACGGCtggatctaccac |
| Clone 7 | gccatcgatgTCAGCGATCGAAACATCGCGtggatctaccac |
| Clone 8 | gccatcgatgAATTACCGCGGGGACCGGCTtggatctaccac |
| Clone 9 | gccatcgatgGATGTGGAGTAAGCCAGTCGtggatctaccac |
