# Supplementary material for: Stable integrant-specific differences in bimodal HIV-1 expression patterns revealed by high-throughput analysis
Source: PLoS Pathog. 2019 Oct 4;15(10):e1007903. doi: 10.1371/journal.ppat.1007903 (PMC6795456; doi:10.1371/journal.ppat.1007903)
Supplement: S2 Table — (PDF) [file ppat.1007903.s008.pdf]

S2 Table: **Integration sites**

zipcode	ins Chrom	ins BP	ins Strand	num Shear Points (3nt Rule)	num Shear Points (unique)
AAACGGTTACAGCGTGGCAC	chr3	18395421	rev	17	48
AAAGCGCCTTACGCGCACGA	chr1	108595225	fwd	15	94
AAATCCACTGTTGTATAATG	chr2	63820806	fwd	27	102
AACACAGGCTGTCATAGCTG	chr17	74702540	rev	20	49
AACAGTGTCTTTGAAAACCC	chrX	118686433	rev	3	3
AACATAAATGGATAGCCATG	chr16	14672056	fwd	9	32
AACTCGTTTACCCCTTCTAC	chr3	126502143	rev	17	24
AAGAATCGCCCCTTGCGCGA	chr12	123700550	fwd	27	73
AAGAGTAAGCAGCAATATA	chr1	194511803	fwd	9	17
AAGCGTCTCAACCAATCGAA	chr15	83807195	rev	14	32
AAGGTAGTCTCCACGAATAT	chr21	33751694	rev	14	29
AATCGGCCTCCTTAACGATC	chr11	68293424	rev	8	13
AATTCTGTAAGTGTGTCGAA	chr2	32110713	rev	34	159
AATTGTTCACCTTTACTGGA	chr3	125254009	fwd	14	34
ACACGAGAAGTTACTTGCTG	chr6	35893422	rev	27	99
ACCCTTGCATAGTATCAGAC	chr4	83805060	rev	11	26
ACCGCGAATGACTTGGACTA	chr14	24691959	rev	15	37
ACCGCGACAAGGGGCTCCTC	chr11	108580222	fwd	12	44
ACGACGTCGAATCGTGATAT	chr10	95445139	rev	13	25
ACGCAACGGGGGACTGGGGC	chr20	35705414	rev	10	14
ACGCGTTACACATGTACGCA	chr2	69552981	fwd	13	19
ACGGAACGCCAATATGCGTG	chr1	59456184	fwd	13	26
ACTGAGTTCCACAGATAGCA	chr16	2299453	fwd	14	46
ACTGCAAAGCGGGCCGACAG	chr12	57457811	fwd	8	10
AGAAATCCCTTAACAGAGAG	chr2	55749153	fwd	7	14
AGAAATCTGATAACTTTAGG	chr1	153869984	rev	4	4
AGAATGAAGGGGAACTGACT	chr17	29592319	rev	1	2
AGACCCTGCGGTCACATCAG	chr12	50917807	fwd	13	40
AGACCTCCTTGGGGAGTCCG	chr11	134304579	fwd	5	11
AGACTGAGAGTCAACGCTTC	chr11	65634277	fwd	5	13
AGAGACAAATTGAAACCGAT	chr3	52295062	rev	21	43
AGATGCCCAAGCAGAAAACT	chr9	88613604	rev	15	34
AGATGTTCGACGAGCAACGT	chr17	4204916	rev	12	50
AGCGACGTATCATAGAGAGG	chr20	43269953	fwd	15	29
AGCGTGACCCTAGTTCTATC	chr15	89804561	rev	21	44
AGCTATTCATGAAGACCTGG	chr19	19234772	fwd	21	62

AGCTTCTGCGTACCCCATAT	chr16	399531	rev	21	67
AGGCCGGGACCGCATCGGGT	chr3	169864928	rev	20	55
AGGGATTCGGTCGCCGATAT	chr3	15754061	rev	16	37
AGGTTCTCATACGAGAACCT	chr11	65046054	rev	21	52
AGTAACCCTTAGTTAGGGTA	chr17	17175509	rev	23	166
AGTTGTCATACGTATGCAGT	chr21	47923541	rev	11	56
ATAAACCCTTGGGAGTATCT	chr15	62283187	fwd	18	28
ATAATTGCTACTCACGGATG	chr1	185088174	fwd	12	26
ATACCGATGCTATTTTTATT	chr1	149731511	rev	7	28
ATACGAGCATCCCCCGGTAC	chr2	128861119	fwd	20	47
ATATCTGGAAACTTCTGTTC	chr11	68337332	rev	15	33
ATATGAGCCGTTTAACAGAG	chr17	7214174	fwd	10	19
ATCATTCTTTCGCAAAAAAA	chr3	195787073	rev	5	6
ATCCATCTAGAATCGGGGAC	chr1	93603537	fwd	18	54
ATCGGTATTGATGTGCAAGC	chr10	5757714	fwd	25	96
ATGGCAACCCTAACCACTAT	chr1	116042595	fwd	18	55
ATGTAGCATTGCATGTATGC	chr3	34152671	rev	10	15
ATTCTCACTTTCTGTCCCGC	chr19	42058337	fwd	26	71
ATTCTGGTTCTTAGGGGACT	chr6	135738933	rev	12	24
ATTGACGCCAAGTGCCAGCG	chr16	66849235	rev	17	49
ATTGGCAGACAGCTAAATCA	chr12	62768615	fwd	21	87
ATTGTAAGGCGCTCTTCCAC	chr22	41328631	rev	3	5
CAAACTAGGGTTTAGGATAA	chr19	13258242	fwd	1	2
CAACGGCCTGTGAGGATGGA	chr1	11730262	rev	21	69
CAACTCCTGCTCCCACCAAT	chr21	45454599	rev	8	13
CAAGCAGTGAAACCGATTAG	chr19	52072025	fwd	21	42
CAATTAAGTGTGAATTGTCG	chr17	58008202	fwd	29	94
CACAATTCTGATTTCCTATC	chr18	59800169	rev	20	68
CACGGGTGATACGTCCTGAA	chr2	160590814	rev	1	1
CACTCTACATCATAAGTAGT	chr7	135073658	fwd	22	67
CACTCTCTATGTCGTGTTAT	chr2	51538264	rev	5	7
CACTGCCCAAGCAATTTCAT	chr6	89600354	fwd	10	13
CACTGTGTAACCCTCGTCGC	chr16	57243954	fwd	19	58
CACTTGCGGTGATCCCTGGG	chr20	52452675	rev	23	196
CAGCACGATAAAATACTCTC	chr2	99326959	rev	2	2
CATATTGACACCCGCTGGCT	chr1	114972800	rev	4	7
CATCCAGACTCTTAGTAACT	chr16	67588223	fwd	20	48
CATCGTAAAGTGAGCGTTAA	chr18	9183245	rev	12	22
CATCTGCGTCCTGCCTCGTC	chr7	43644182	fwd	13	24
CATTAATTTACGAAGGTTTA	chr17	47727743	rev	2	3

CATTTGTTGACCAGCAGCGT	chr5	83921932	fwd	10	15
CCAACGGAATTGCCCTTTCC	chr3	125764667	rev	17	51
CCACAGTTCACCCTTTGTAA	chr3	194120775	rev	1	1
CCCAATTATACTCAGGGCGA	chr20	21336740	fwd	10	17
CCCCACGGGTTCAGCTGATT	chr3	149585548	fwd	7	16
CCCCATACCTGTACTCCGCC	chr2	26596975	rev	9	12
CCGGCAAAGTCACCACCACT	chr2	160590801	rev	18	102
CCGGGCATGATCGATAATGT	chr17	60584939	fwd	6	13
CCGTAATCATGATCTAGTCC	chr4	57288570	fwd	18	39
CCTCAAGCGGAAATTGGAGG	chr2	242230256	rev	12	19
CCTCCCCTTAAGTGGGGATG	chr2	128237892	fwd	24	65
CCTCGATTCGAGAATTCGTG	chr1	213036048	fwd	19	85
CGAGCATTTTTGAATGGTTG	chr5	138661871	fwd	22	45
CGAGTGTATCATCACTCGAA	chr11	47082155	fwd	12	36
CGATGGAAAAAATGACGTTA	chr12	101744563	rev	20	40
CGCAGTAGTATGAAGTATGA	chr20	34247250	rev	24	106
CGCATGGGGCGGGAGCAGAG	chrX	70508662	rev	2	2
CGGAAAAAAAAGAGGCCGGG	chr5	132045324	rev	21	127
CGGACCCACTTGCTTCACGC	chr1	31465909	fwd	17	28
CGGAGACAACTCTTCTGTTT	chr21	47905470	rev	10	18
CGGGACTCTGTTTCGCAAAC	chr21	23801760	rev	8	14
CGGGCCGGGCACCTACGCAG	chr12	49074757	fwd	10	27
CGGTTATCTTTCCGGCTCTA	chr17	32704009	rev	14	39
CGGTTTGCGGGGTCGCCTAC	chr10	53467887	rev	13	24
CGTCGAGTGCACGAAGCACA	chr16	1696834	fwd	23	64
CGTCTCACTGAGCAGCCAAG	chr20	43666534	fwd	10	23
CGTGATTTGTTGTAAGATGA	chr13	50649083	rev	7	13
CGTTATATGATCGATCATAT	chr4	40112573	fwd	17	58
CTCATTTTAGCTATGTCCGT	chr5	31521263	rev	11	31
CTCCCCCGCACAATAGTGTG	chr17	47405396	fwd	11	50
CTCGTGATGCCCGTAGGAGC	chr17	78596696	fwd	1	1
CTGATATAGCTATAGAGTGA	chr15	91341982	fwd	4	8
CTGCCGAAGTATATCAGCAG	chr22	40863064	fwd	5	20
CTGCGGATTAGCGTCTGTAA	chr1	114982359	rev	8	12
CTGCGGTGTGGTTCTTGGCA	chr15	77438425	rev	8	16
CTGCTCGTATAAAGTAGTAC	chr21	38872071	fwd	7	14
CTGGAAGAACTTTTGCAGCA	chr11	33958369	fwd	1	1
GAAAAGTTTGCCCACAATAC	chr13	60681242	rev	16	24
GAAACTCTGCACCTATTAAT	chr18	20598740	rev	15	25
GAACAGGCTAAGTACTCCCT	chr17	30682805	rev	19	67

GAAGACACATTAACTTGTAC	chr14	106458885	rev	16	25
GACATACTAGCGCGGCTAT	chr1	149850614	fwd	24	111
GACCTATGCGTATTGCGGCA	chr9	131725938	fwd	21	59
GACGACAACCACCGACCGAC	chr9	15725780	rev	19	52
GACGCTCCAAGATTCTTAAC	chr3	150910355	rev	18	123
GAGCGGCGAAATCGGACCTG	chr9	132775655	fwd	6	12
GAGTCGTAGGTATTAGCGAA	chr17	74082586	fwd	24	65
GATAAAACGAGCATTAGAAA	chr5	176681791	fwd	10	21
GATAGCCCGTAGGCGACGTC	chr1	156314957	fwd	15	23
GATGTCTGAGGCCGTACAAT	chr17	4188572	rev	5	24
GCACATAAAGAAGTGCTTTG	chr13	99864323	rev	6	20
GCACATCACGCGCAGCGGGC	chr7	23643901	rev	8	16
GCACCCCGGGAGTGAAAACC	chr12	48443290	rev	14	25
GCAGGGTAGCAACATAGGCC	chr17	27837380	fwd	17	200
GCATCTCTCCCGTAACCGCT	chr17	64798415	rev	16	26
GCATTCGTCGCCTTTTTCGA	chr6	147631249	fwd	5	11
GCCACTTACATCTAGAAAGC	chr6	111025400	rev	5	5
GCCATGTGGCGCGATATACC	chr3	151072823	fwd	10	37
GCCCATCCGGTCCGGAGATC	chr15	72838284	fwd	13	29
GCCCCAAAGGGACTGAATCC	chr4	48876058	rev	4	5
GCCCCGACCCGCGAGGGGGA	chr7	44689340	rev	16	46
GCCTGACTCATATTCAGGGA	chr19	18431133	fwd	26	110
GCCTTGATTGTAAGGTGGGG	chr2	105929621	rev	7	13
GCGACCCCCAAGAACCAGAT	chr7	72402060	fwd	16	33
GCGACGCTTGACAATGTCGA	chr4	83782207	fwd	26	91
GCGTCAAGAAATAGAGAATG	chr8	86106668	rev	13	31
GCGTCATGGAGTTCCTCCAT	chr16	3027720	fwd	24	166
GCGTCCATCAGCACATCAGG	chr19	50292416	fwd	11	26
GCGTGAACATTATACACTTT	chr1	152355191	fwd	27	103
GCGTGCGGTTGCTACACAGA	chr1	168154114	rev	11	21
GCGTTAGTTAGCTCGGTTGG	chr9	131819215	rev	19	43
GCTACAGATGTTCTTGCCGC	chr11	62400130	rev	2	2
GCTGTTTCTGGGGCGCTCA	chr1	51922203	fwd	9	17
GCTTAAATCGACAAGCGTAT	chr12	25484506	fwd	16	38
GGAGACCGCAATCCTACGCG	chr2	26026282	rev	5	9
GGCACCGGGGCAATCCGGCA	chr20	47890056	fwd	6	9
GGCCCCTGATGCTTGGATTT	chr4	184917808	rev	6	7
GGCCGTAAAGGGCTTAAATG	chr7	92200060	rev	17	38
GGCTAGAGTAGCCGATCGAG	chr6	97712715	rev	11	22
GGGATTCTGGACACTAGCCG	chr2	61515437	fwd	5	6

GGGCACTAGGCCGATAGTTT	chr5	52074929	fwd	3	3
GGGCATTACATCTAATCAAA	chrY	23350488	fwd	17	38
GGGGAACCCCCGCGAGGGGA	chr9	96241196	fwd	2	2
GGGGCTTCAAATCACGCAAT	chr4	69197416	rev	19	48
GGTAAAGTTACATCGGATAC	chr9	15725794	rev	1	1
GGTACATATCCCAAAAGGGA	chr12	102528184	fwd	14	62
GGTGATTCGGTTATGTGCTA	chr1	186305474	rev	18	44
GTACAAGCTTTGTAACCCGA	chr7	99079633	fwd	14	35
GTATTCTCGCACCACCCATC	chr19	10267306	rev	21	54
GTCGGATATAAATCCGCTCT	chr10	27413350	rev	15	22
GTGGTAAAGTAAAACTTGGG	chr16	14583042	rev	11	23
GTGTCGTTAAGCCCGGCATA	chr12	57627963	rev	23	74
GTTAGGTCACTAACAACGGA	chr7	24684959	fwd	6	8
GTTCGCTTCTGGTTCGTGT	chr10	116595096	fwd	17	26
GTTTATCTGGAAGCGTACTC	chr15	78338420	fwd	2	3
TAACAGTATTTTCTAGGAAC	chr14	74139316	rev	9	13
TAATGTTAGGTAATTCCGGA	chr2	20509207	rev	5	7
TAATTGCATAAATCTCGCCG	chr2	192268452	fwd	8	22
TACCTACATGCTTCGCCGTA	chr12	49816602	fwd	16	43
TACGGACAGGTTTAGACCTA	chr11	108219643	fwd	16	32
TACGTTCCGCATAAACCGTC	chr17	7243712	rev	17	27
TACTGAAGATCTAGTCATGA	chr5	32547909	fwd	27	97
TAGAACCCCAATCCGGGGCC	chr3	196348422	rev	27	110
TAGTGTGCTAGGTACTTCCC	chrX	13757756	rev	8	12
TATAATGTTTAGTCGCCTGG	chr6	37905815	fwd	27	109
TATACAGCCCGAACACAATG	chr6	121609102	rev	6	8
TATCTCCCGCATTATGTCGC	chr2	9666113	rev	18	46
TATCTTAAATAGTTTTTATA	chr7	7264648	rev	6	14
TATGGTAGGATGCTCAACAC	chr4	71573510	rev	15	65
TCAGACCCCCGAAATGGGCC	chr16	69186487	rev	16	73
TCATACTCAAGACAGCCGAT	chr17	65911250	fwd	25	131
TCCATTGGAGCCCAAGTGTT	chr3	50091269	fwd	11	26
TCCGAGGCGACGCCTTTCGG	chr12	49069223	rev	27	140
TCCGATCCAAGCATAAAAGA	chr1	1716276	rev	25	79
TCCGTCCGGTATTTAGATTT	chr11	65870281	rev	11	20
TCGAAACAGAGTTTGTAGAG	chr3	182913103	fwd	20	129
TCGGCAGCCCCCTTCGAATG	chr12	49429664	rev	13	40
TCGGCGGGTACAACCAATGA	chr7	135273281	fwd	16	25
TCGTAGGACAGTCGAGTTTG	chr3	152006563	fwd	8	16
TCGTGGTACCGAACGACTAG	chr17	3723709	rev	15	31

TCTCACCCCCTTGAATACAG	chr1	114440807	rev	29	110
TCTCGGTACTAGGGGGCCTT	chr6	109436863	rev	17	111
TCTTACACGGATTAGTGCAA	chr17	2026530	fwd	23	119
TGAAACCTGACGAAACTGTG	chr15	57245476	rev	11	24
TGAACCGCGGATTTTGGGCG	chrY	2825026	rev	12	24
TGACCTCCGATCACCCGGAG	chr20	62246791	fwd	12	36
TGAGAGATCGCTACACTGTA	chr3	196291350	rev	29	171
TGCAATATCATGGCGGTTGC	chr1	26287657	rev	11	42
TGCCCTTGTGTGTCTGATAT	chr17	55342783	rev	12	35
TGCCTAGAGCATACCTTAAG	chr17	79812870	fwd	4	7
TGCGCCCGATTTCTATCAAA	chr12	50989965	fwd	12	18
TGGAATCGCGCGGTATACCA	chr4	74067540	fwd	5	13
TGGACACTTGGTTCCTAGTG	chr22	41263568	fwd	9	12
TGGGATATCACTAACGATCC	chr7	102836180	fwd	15	38
TGGTCTATCAGAGCGATATG	chr14	56106865	fwd	5	13
TGTATCAGCTTGATATCTTT	chr2	128252115	rev	10	17
TGTGAGCGGAGTCATCCGCG	chr1	16635538	rev	19	41
TTCAGAAATTCAACCGCATC	chr18	12959748	fwd	21	40
TTCATGCAGCGGGGGTACAA	chr2	240209559	rev	28	98
TTCGATAAACGCAGACTATT	chr4	154397977	rev	19	37
TTGCACGGCATGTTACATTT	chr5	138682146	fwd	18	35
TTGCCTAGATCGCCGCTTCT	chr9	134275447	fwd	17	50
TTGCTGTCCCGCTAGCGAGC	chr6	135481600	rev	15	45
TTGCTTACGAACTCAGTCTC	chr17	57165969	rev	21	108
TTGGAAGAGGGTACTTGGAA	chr22	36237160	rev	10	12
TTTAGTGACGTGAGGATCAC	chr19	2148971	fwd	15	59
TTTATTGCCCGACCACAATT	chr16	66837004	fwd	10	23
TTTCTCTAACCAACAATCCG	chr19	58337901	rev	9	18
TTTGAGGGGTACTACACCGC	chr6	42633342	fwd	6	9
